# Genomic resources for the Neotropical tree genus *Cedrela* (Meliaceae) and its relatives

**DOI:** 10.1186/s12864-018-5382-6

**Published:** 2019-01-18

**Authors:** Kristen N. Finch, F. Andrew Jones, Richard C. Cronn

**Affiliations:** 1 Department of Botany and Plant Pathology, Oregon State University, Corvallis, Oregon 97331 USA; 2 Smithsonian Tropical Research Institute, Balboa, Ancon, Republic of Panama; 3 USDA Forest Service Pacific Northwest Research Station, Corvallis, Oregon 97331 USA

## Abstract

**Background:**

Tree species in the genus *Cedrela* P. Browne are threatened by timber overexploitation across the Neotropics. Genetic identification of processed timber can be used to supplement wood anatomy to assist in the taxonomic and source validation of protected species and populations of *Cedrela.* However, few genetic resources exist that enable both species and source identification of *Cedrela* timber products. We developed several ‘omic resources including a leaf transcriptome, organelle genome (cpDNA), and diagnostic single nucleotide polymorphisms (SNPs) that may assist the classification of *Cedrela* specimens to species and geographic origin and enable future research on this widespread Neotropical tree genus.

**Results:**

We designed hybridization capture probes to enrich for thousands of genes from both freshly preserved leaf tissue and from herbarium specimens across eight Meliaceae species. We first assembled a draft de novo transcriptome for *C. odorata,* and then identified putatively low-copy genes. Hybridization probes for 10,001 transcript models successfully enriched 9795 (98%) of these targets, and analysis of target capture efficiency showed that probes worked effectively for five *Cedrela* species, with each species showing similar mean on-target sequence yield and depth. The probes showed greater enrichment efficiency for *Cedrela* species relative to the other three distantly related Meliaceae species. We provide a set of candidate SNPs for species identification of four of the *Cedrela* species included in this analysis, and present draft chloroplast genomes for multiple individuals of eight species from four genera in the Meliaceae.

**Conclusions:**

Deforestation and illegal logging threaten forest biodiversity globally, and wood screening tools offer enforcement agencies new approaches to identify illegally harvested timber. The genomic resources described here provide the foundation required to develop genetic screening methods for *Cedrela* species identification and source validation. Due to their transferability across the genus and family as well as demonstrated applicability for both fresh leaves and herbarium specimens, the genomic resources described here provide additional tools for studies examining the ecology and evolutionary history of *Cedrela* and related species in the Meliaceae.

**Electronic supplementary material:**

The online version of this article (10.1186/s12864-018-5382-6) contains supplementary material, which is available to authorized users.

## Background

*Cedrela* P. Browne is a Neotropical tree genus in the mahogany family (Meliaceae Juss.) that consists of 18 named species [1]. *Cedrela* species range from Mexico (24° N) to Argentina (27° S), and many species are sympatric across their geographic distributions. Three *Cedrela* species, *C. angustifolia* DC., *C. fissilis* Vell., and *C. odorata* L., are listed with the Convention on International Trade in Endangered Species of Wild Fauna and Flora (CITES) under Appendix III, which requires documentation showing that imports did not originate in parts of their range where logging is prohibited, namely Peru, Guatemala, Bolivia, and Brazil [2]. Large and overlapping species ranges complicate the identification of imported *Cedrela* logs and processed timber [3], as the task of distinguishing between restricted versus legally logged *Cedrela* species falls to customs officials who often employ the expertise of a wood anatomist [4]. Distinguishing between restricted and legal stands of the same species, for example *C. odorata* harvested in Ecuador (legal) versus *C. odorata* harvested in Peru (where CITES prohibits logging), presents and even greater challenge because wood anatomy alone cannot identify and differentiate source populations of the same species [4].

Genetic identification approaches can be used to supplement wood anatomy for the taxonomic and source validation of imported timber [5–7]. However, three challenges exist for the development of genetic markers for identification of *Cedrela* wood specimens. First, it is uncertain whether the 18 described *Cedrela* species represent distinct species, subspecies, or hybrids [8]. The most-recent DNA-based phylogenies for *Cedrela* show inconsistent support for some species [1, 8, 9]. Moreover, *Cedrela* are tetraploid [1, 10, 11] and evidence suggests that common DNA markers like nuclear rDNA used to infer the phylogeny of *Cedrela* may reflect concerted evolution and non-Mendelian inheritance, rather than evolutionary relatedness [12]. Because the *Cedrela* phylogeny forms the basis for CITES protection, taxonomic uncertainty complicates the enforcement of CITES regulations for logging and trade in *Cedrela* timber. The second challenge pertains to the development of markers for geographic assignment of *Cedrela* specimens to population of origin. Spatial genetic structure among populations of *C. odorata* and *C. fissilis* [8, 9, 13–17] has been examined using multiple genetic markers (nuclear rDNA, chloroplast non-coding genes, chloroplast microsatellites), and these studies identified spatial structure across broad geographic regions: a latitudinal gradient in chloroplast and nuclear haplotypes for *C. odorata* populations across the extent of its range [8], and a longitudinal gradient among *C. fissilis* flanking the ‘Cerrado’ savanna ecoregion of Brazil and Bolivia [13, 14]. While these markers may be useful for coarse-level regional assignment, it is unclear if they could provide fine-scale classification accuracy necessary for differentiating timber from protected concessions, or differentiating timber from neighboring countries that offer contrasting levels of protection for *Cedrela* species. The third challenge for the genetic identification of *Cedrela* timber is a lack of specimens of known geographic origin which are necessary for the construction of a comprehensive reference genomic database for taxonomic and source validation of confiscated timber.

This study addresses the above challenges by establishing a foundation of taxon-specific genetic information via high-throughput genomic sequencing of georeferenced herbarium specimens. We designed a set RNA probes for hybridization capture target enrichment (‘target capture’) of 10,001 putative low-copy gene targets from the *Cedrela odorata* transcriptome, and used these probes to enrich targets from genomic libraries of five *Cedrela* species, including the CITES-listed species, to identify high-confidence single nucleotide polymorphisms (SNPs) for species identification [18–20]. We also evaluated the transferability of enrichment probes for the genomes of three other Mahogany family relatives, including American mahogany (*Swietenia mahagoni* (L.) Jacq.; subfamily Cedreloideae), *Guarea guidonia* (L.) Sleumer, and *Trichilia tuberculata* C. DC. (subfamily Melioideae), and we determined the number of genes that could be enriched to a usable depth for each species. Finally, we provide draft chloroplast genomes for 43 specimens representing the eight evaluated taxa. These genomic resources can be used to provide more accurate estimates of taxonomic boundaries, population genetic structure, and historical demography for *Cedrela* species, and these may aid the assessment of species risk and forest resource conservation. The generality of these techniques (probe design, assessment of probe transferability, target capture efficiency) has wide applicability for the development of tools to combat illegal logging across tropical hardwoods, and the markers we report herein will be useful for developing protocols for genotyping of *Cedrela* wood.

## Results

### Reference transcriptome and capture probe design

**Fig. 1:**
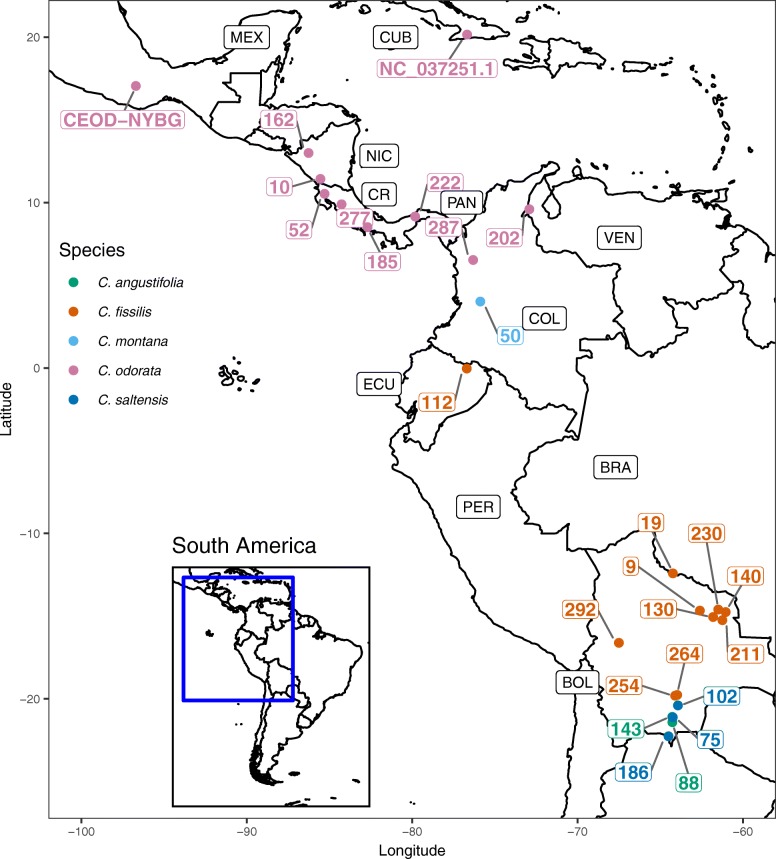
Map shows a portion of Central, South America, and the Caribbean where CEOD-NYBG (approximate location) and the *Cedrela* specimens originated. We also include the location of origin for *C. odorata* NC_037251.1. Specimens are coded with the number that matches their Specimen ID (see Additional file 1: Table S1) and color coded by species. Other labels correspond to relevant country codes: MEX (Mexico), CUB (Cuba), NIC (Nicaragua), CR (Costa Rica), PAN (Panama), COL (Colombia), VEN (Venezuela), ECU (Ecuador), PER (Peru), BOL (Bolivia), and BRA (Brazil). Base map shapefiles were obtained from the World Borders Dataset http://thematicmapping.org/downloads/world_borders.php

A single *Cedrela odorata* leaf from Oaxaca, Mexico obtained from the New York Botanical Garden (CEOD-NYBG) was used for transcriptome reference assembly and target capture probe design (Fig. 1; see Additional file 1: Table S1). We extracted RNA and DNA from fresh tissue from a mature, expanded leaflet, and poly(A)-selected RNA was converted into cDNA and sequenced using the Illumina HiSeq 3000 platform. Detailed information about the transcriptome is provided in Table 1. RNA-seq yielded 1.5 × 10^8^ paired end 101 base pairs (bp) sequences (30.4 Gbp), and quality trimming and adapter filtering removed about 4% (6.0 × 10^6^) of these paired sequences. From the trimmed sequences, one-third (4.9 × 10^7^) could be combined to form FLASH-extended ‘super reads’ [21], and these were combined with unextended paired trimmed reads for de novo transcriptome assembly using a pipeline [22] based on the ABySS assembler ([23]; see Methods). Our assembled transcriptome contained 52,181 transcripts (‘gene models’) from 4.8 × 10^7^ nucleotides [24]. Of these, 28% (14,508) of gene models were greater than 1000 bp (Kbp) in length, and they range in length from 37,635 bp to 200 bp (arbitrarily assigned length cut-off). The estimated mean gene model length was 916 bp, the N50 was 1470 bp, and overall transcript GC content was 39.5%.

**Table 1:** *Cedrela odorata* RNA sequence yield after various quality filtering and optimization steps, and final transcriptome summary statistics

*Raw Reads*	Number of sequences	Minimum length	Maximum length	Sum of bases	Average Length
Read pairs	150,329,597	101	101	15,183,289,297	101
*Read Trimming and Quality Filtering* via *Trimmomatic*					
Read 1	144,314,023	36	101	14,516,997,898	100.6
Read 2	144,314,023	36	101	14,401,698,529	99.8
Sequences removed	6,015,574			666,291,399	
*Read Extension* via *FLASH*					
Un-extended read 1	95,727,746	36	101	9,626,867,686	100.6
Un-extended read 2	95,727,746	36	101	9,514,449,431	99.4
Super reads	48,586,277	36	192	7,845,887,083	161.5
*Final Assembly*					
CEOD-NYBG Transcriptome	52,181	200	37,635	47,811,997	916.3

All lengths are in base pairs

Gene family and ontology prediction with TRAPID/PLAZA 2.5 [25, 26] determined that 73% (38,218) of the gene models contained open reading frames (ORFs) and a stop codon, and that 49% (25,755) of gene models included a start codon. Full-length annotations were determined for 14% (7165) of gene models, quasi full-length annotations (longer exons than predicted) were inferred for 18% (9540) of the gene models, and partial–length annotations were provided for 28% (14,491) of the gene models. The mean length of ORFs among our gene models was 418 bp. TRAPID/PLAZA inferred that 21% (10,904) of gene models show one or more putative frameshifts. Forty percent (20,985) of gene models could not be annotated. More than half of our gene models could be associated with a gene family (31,512; 60.3%), and 6043 gene families were represented among our gene models. The largest gene family identified (5860_HOM000003) included 304 transcripts and showed homology to pentatricopeptide repeats (PPR), a high-copy gene family known to coordinate nuclear control of organelles and organelle signaling [27]. TRAPID/PLAZA predicted over 5000 total gene ontology terms were represented in our transcriptome reference (2.6 × 10^4^ gene models with gene ontology terms; 49% of gene models). Moreover, 54% (2.8 × 10^4^) of our gene models could be associated with protein domains curated in InterPro [28].

We designed probes for target capture from 10,001 gene models that appeared to be present in low-copy number in the genome of CEOD-NYBG. We identified the relative abundance of each of the 52,181 gene models by mapping truncated genomic DNA sequencing reads (50 bp) to the CEOD-NYBG transcriptome with BBTools [29]. This allowed us to count the number of genomic reads homologous to each transcript; these values were normalized by transcript length to reflect mapped reads per 1 Kbp of gene model (or “RPK”). The RPK values for genomic regions homologous to reference transcripts showed a global mean of 3.5, and ranged from 0 (presumably contaminants, e. g. rare leaf endophytes) to 1890 (presumably expressed, high copy genes). Gene models were sorted in ascending order by RPK value, and we selected 10,001 gene targets with ranks between 5000 to 15,000 (RPK range: 0.6 to 1.0; mean RPK = 0.8). Our gene targets ranged in length from 200 bp to 8118 bp with an estimated mean length of 576 bp. See Additional file 1: Figure S1 to examine the distribution of mapped reads for the transcriptome and RPK range of values for our selected gene targets.

### Target capture

We assessed the transferability and target capture efficiency of hybridization probes designed from the CEOD-NYBG reference tree using 8 specimens of the source species (*C. odorata* L.), 16 specimens from four other *Cedrela* species (*C. angustifolia* DC., *C. fissilis* Vell., *C. montana* Moritz ex Turcz., and *C. saltensis* M. A. Zapater & del Castillo), 2 specimens from the same subfamily in the Meliaceae (*Swietenia mahagoni*), and 17 specimens from a different subfamily in the Meliaceae (*Guarea guidonia*, *Trichilia tuberculata)*. DNA sequencing after target capture generated 3.7 × 10^8^ paired reads (73.9 Gbp), with a mean per-library yield of 8.5 × 10^6^ paired reads (range: 4.4 × 10^5^–2.9 × 10^7^ paired reads; Table 2).

**Table 2:** Sequencing metrics for *Cedrela* and Meliaceae samples

Specimen	SRA Accession	Sequence Yield	On-Target Yield	On-Target (%)	Chloroplast (CP) Yield	CP (%)	CP Depth	CP (%) Covered	‘N’ Bases (%)
*C. angustifolia* 88	SRS3532111	1,515,526	488,207	32.2	248,243	16.4	157	100.0	0.1
*C. angustifolia* 143	SRS3532109	8,462,020	3,191,296	37.7	901,138	10.6	568	100.0	0.1
*C. fissilis* 9	SRS3532073	1,174,195	286,398	24.4	93,257	7.9	59	99.9	0.2
*C. fissilis* 19	SRS3531955	5,431,374	2,122,651	39.1	333,680	6.1	210	99.9	0.2
*C. fissilis* 112	SRS3532019	1,750,937	376,881	21.5	151,391	8.6	96	99.8	0.4
*C. fissilis* 130	SRS3532093	5,351,355	2,154,295	40.3	135,883	2.5	86	99.9	0.2
*C. fissilis* 140	SRS3532018	12,183,129	5,047,225	41.4	403,902	3.3	255	100.0	0.1
*C. fissilis* 211	SRS3532001	1,272,124	259,059	20.4	167,826	13.2	106	100.0	0.2
*C. fissilis* 230	SRS3532122	3,251,712	1,183,526	36.4	106,353	3.3	67	99.9	0.4
*C. fissilis* 254	SRS3532102	17,394,724	5,583,057	32.1	643,227	3.7	405	100.0	0.1
*C. fissilis* 264	SRS3532060	19,290,108	7,776,152	40.3	356,979	1.9	225	100.0	0.1
*C. fissilis* 292	SRS3532121	7,116,518	2,752,495	38.7	1,866,075	26.2	1176	100.0	0.1
*C. montana* 50	SRS3532121	5,561,468	2,026,840	36.4	577,438	10.4	364	100.0	0.2
*C. odorata* 10	SRS3532068	4,009,101	1,472,131	36.7	273,812	6.8	173	99.8	0.4
*C. odorata* 52	SRS3532030	12,254,507	4,791,273	39.1	618,594	5.0	390	100.0	0.1
*C. odorata* 162	SRS3531967	8,942,300	3,574,795	40.0	756,725	8.5	477	100.0	0.1
*C. odorata* 185	SRS3532120	5,713,214	2,177,026	38.1	382,876	6.7	241	100.0	0.1
*C. odorata* 202	SRS3532089	6,372,915	2,366,009	37.1	116,791	1.8	74	99.7	0.4
*C. odorata* 222	SRS3532005	10,421,206	4,017,316	38.5	289,695	2.8	183	99.8	0.3
*C. odorata* 277	SRS3531957	6,794,076	2,539,070	37.4	309,154	4.6	195	99.8	0.5
*C. odorata* 287	SRS3532135	438,310	6566	1.5	35,604	8.1	25	90.2	7.7
*C. saltensis* 75	SRS3532020	29,081,506	10,692,874	36.8	831,948	2.9	524	100.0	0.1
*C. saltensis* 102	SRS3532010	5,252,123	2,107,682	40.1	672,165	12.8	424	100.0	0.1
*C. saltensis* 186	SRS3532110	1,829,249	554,830	30.3	125,974	6.9	79	100.0	0.2
*G. guidonia* 2	SRS3532153	5,211,243	68,501	1.3	92,122	1.8	78	74.2	19.5
*G. guidonia* 4	SRS3532152	6,710,368	100,652	1.5	129,500	1.9	105	77.8	13.9
*G. guidonia* 7	SRS3532151	6,850,155	101,252	1.5	81,731	1.2	74	69.8	17.8
*G. guidonia* 9	SRS3532150	14,213,046	206,827	1.5	197,446	1.4	139	89.3	13.5
*G. guidonia* 10	SRS3532124	7,833,412	91,070	1.2	69,582	0.9	69	63.9	15.0
*G. guidonia* 11	SRS3532123	9,166,583	123,598	1.3	279,357	3.0	194	90.9	7.3
*G. guidonia* 13	SRS3532149	9,909,255	133,845	1.4	259,749	2.6	184	88.9	7.6
*G. guidonia* 15	SRS3532148	10,634,632	148,498	1.4	267,334	2.5	185	91.1	7.6
*G. guidonia* 17	SRS3532147	10,900,380	128,635	1.2	138,537	1.3	108	80.8	13.3
*G. guidonia* 19	SRS3532146	16,194,976	195,256	1.2	342,985	2.1	229	94.5	8.3
*S. mahagoni* 21	SRS3532156	16,927,562	1,051,671	6.2	1,530,129	9.0	966	99.8	1.5
*S. mahagoni* 22	SRS3532155	18,098,858	1,113,548	6.2	3,806,990	21.0	2399	100.0	0.5
*T. tuberculata* 1	SRS3531991	3,823,599	39,222	1.0	54,256	1.4	159	89.8	17.1
*T. tuberculata* 3	SRS3531995	5,915,718	60,793	1.0	92,170	1.6	76	76.4	14.2
*T. tuberculata* 6	SRS3531996	6,159,511	63,116	1.0	140,155	2.3	110	80.0	11.4
*T. tuberculata* 8	SRS3531994	6,595,654	70,395	1.1	59,560	0.9	50	74.4	23.5
*T. tuberculata* 12	SRS3531993	11,354,222	92,063	0.8	283,424	2.5	56	60.9	8.3
*T. tuberculata* 18	SRS3531989	10,145,721	103,682	1.0	225,870	2.2	196	91.3	12.2
*T. tuberculata* 20	SRS3531990	8,374,551	91,146	1.1	117,705	1.4	90	82.5	13.2

Metrics include: SRA Accession number; total sequence yield (in bp), sequenced reads with identity to the gene target (‘On-Target Yield’, in bp); percent of sequenced reads with gene target identity (‘On-Target (%)’); sequenced reads with chloroplast identity (‘Chloroplast (CP) Yield’, in bp); percent of sequenced reads with CP identity (‘CP (%)’); depth of coverage across the CP reference genome (‘CP Depth’); the percentage of the CP reference genome covered at 1X depth (‘CP [%] Covered’); and the percentage of ‘N’ bases in the specimen chloroplast genome (‘N’ Bases [%])

Sequence reads from individual libraries were mapped to the gene targets to determine the proportion of sequenced reads that were on-target. On average across specimens, 1.6 × 10^6^ sequence reads mapped to the gene targets (mean on-target yield; range: 6.6 × 10^3^–1.1 × 10^3^), or on average, 20% of sequenced reads from an individual specimen mapped to the gene targets (range 0.8–41%). Out of 10,001 gene targets, only 206 (2%) showed no mapped reads. Across all samples, *Cedrela* specimens had a significantly higher proportion of on-target reads (mean on-target yield = 2.8 × 10^6^ reads) than was observed for other Meliaceae genera combined (mean on-target yield = 2.1 × 10^5^ reads; unpaired *t* test, *t* = 4.95, df = 23.86, *p* < 0.001), indicating that hybridization probes derived from *C. odorata* were far more effective at capturing sequence targets from other *Cedrela* than more distant relatives.

**Fig. 2:**
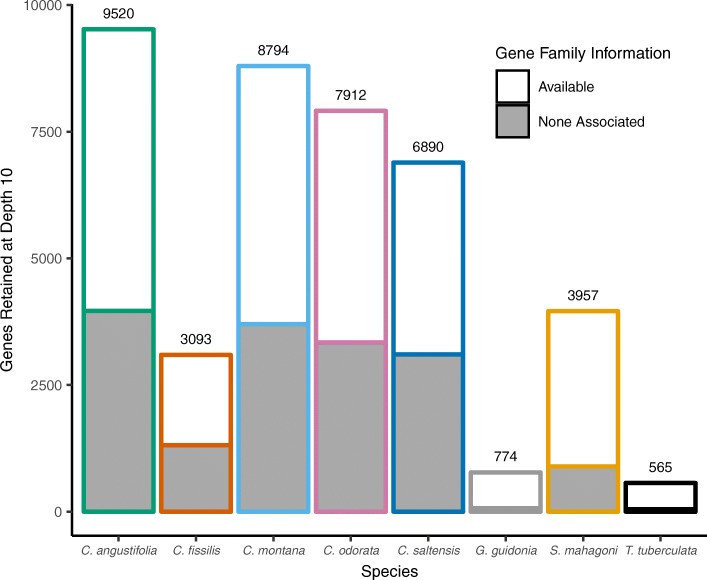
Counts of genes retained after we imposed a depth of coverage threshold of 10X across all specimens for each species group. Retained gene counts are positioned above each species bar. Bar outlines are color coded by species and for *Cedrela*, correspond to Fig. 1. Bar fill indicates the proportion of gene targets associated with gene family information from TRAPID/PLAZA 2.5

To estimate the number of gene targets that could be potentially examined for each species with target capture, we tallied the number of gene targets covered at an average depth > 10 for all individuals in the species (a conservative depth for distinguishing homo- vs. heterozygosity at a locus [18]). As noted above, *Cedrela* specimens yielded the largest number of usable gene targets, with more than 3000 gene targets recovered at a depth > 10 for all *Cedrela* species (Fig. 2). The number of usable gene targets was negatively correlated with the number of samples in each species group, due in part to our stringent depth requirement, sampling variability, and missing information across such a large number of gene targets. Moreover, the number of usable gene targets was dependent upon the quality of the included libraries; for example, *C. fissilis* 112, 211, and 9 showed significantly lower on-target yield compared to other *C. fissilis* libraries (Table 2). This reduced our estimate of reliably enriched genes for this species. *Swietenia mahagoni* showed the highest individual mean depth of non-*Cedrela* genera, and had nearly 4000 gene targets retained at depth 10X or greater. Finally, only 774 and 565 gene targets met our depth filter of 10X for the more distantly related taxa *G. guidonia* and *T. tuberculata*, respectively (Fig. 2).

**Fig. 3:**
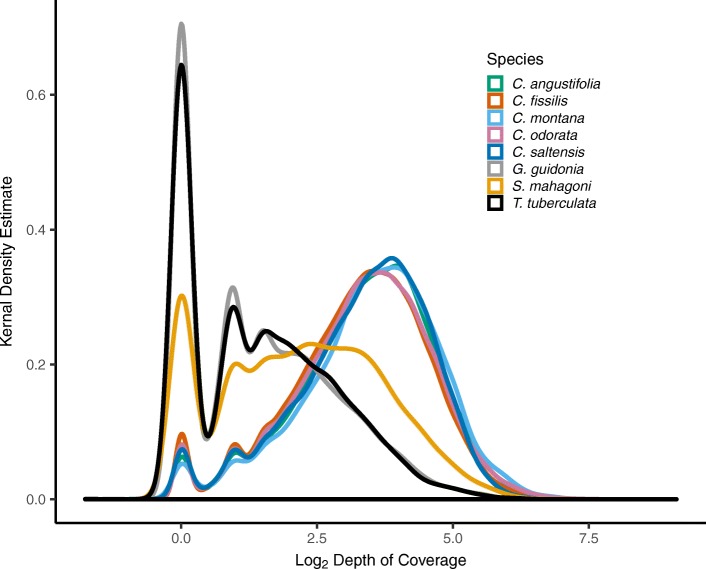
Density plot showing depth of coverage (log_2_ scale) distribution across enriched targets for each species. Species distributions are color coded

The overall proportion of mapped reads to nuclear gene targets is influenced by many factors, such as organelle abundance, library quality, and PCR dynamics, so we compared the efficiency of target capture across species with reduced bias by mapping 10^6^ organelle-depleted reads from each library to the gene targets and estimating resulting read depth. In this exercise, target depth ranged from 0X (at 272 gene targets) to 86.5X, and the estimated mean target depth was 7.5X (1st quantile 3.3X; 3rd quantile 10.0X). The global mean depth across *Cedrela* specimens (12.8X) was higher than the mean depth for *S. mahagoni* (3.4X), *G. guidonia* (0.8X), and *T. tuberculata* (0.6X) specimens. This finding shows that our hybridization probes more effectively sampled *Cedrela* libraries after accounting for differences due to library size and organelle genome proportion (unpaired *t* test, *t* = 26.92, df = 31.65, *p* < 0.001). Figure 3 shows the distribution of depths of coverage for each gene target – individual pair grouped by species (see Additional file 1: Figure S2 for another view of the same data). The distribution for the *Cedrela* specimens are overlapping and converging on mean depth of coverage despite differences in sample size (number of specimens) for each species group and despite variability in on-target yield.

### SNP identification and high differentiation polymorphisms for *Cedrela* species

By using a pipeline combining SAMtools [30] and the Genome Analysis Toolkit [31], we identified 444,979 variants across 23 individual captured libraries from 5 *Cedrela* species. Of the detected variants, 399,117 (89.7%) were bi-allelic SNPs, 28,069 (6.3%) were insertions or deletions (indels), and 17,793 (4.0%) were SNPs with more than two alleles. After removing indels and SNPs with more than two alleles, missing information for bi-allelic SNPs ranged from 7.7 to 35.9% per individual and averaged 19.8%. After applying three stringent filters (biallelic SNPs with minor allele frequency (MAF) > 5%; site quality > 500; no missing information), we detected 119,020 SNPs across 9598 gene targets in our sample of 23 *Cedrela* specimens.

**Fig. 4:**
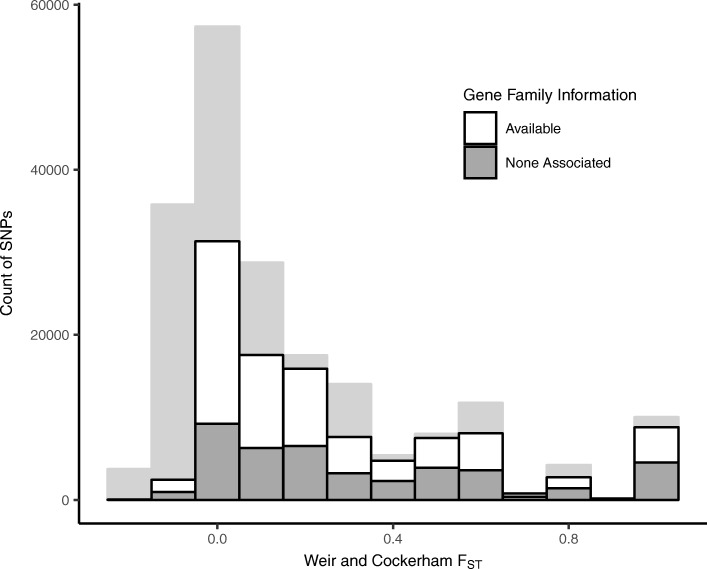
Weir and Cockerham’s *F*_*ST*_ distribution for SNPs identified in *Cedrela* specimens. Light grey bars show the distribution of biallelic SNPs with no missing information. Bars outlined in black indicate biallelic SNPs with no missing information, MAF ≥ 5%, and quality ≥500. Bar fill for bars outlined in black indicates the proportion of SNPs that were found on gene targets that could be associated with gene family information form TRAPID/PLAZA 2.5 as in Fig. 2

To evaluate the utility of these SNPs for species determination, we calculated Weir and Cockerham’s *F*_*ST*_ [32] on a per-marker basis using VCFtools [33], and *Cedrela* species as ‘populations’. We excluded *C. montana* because SNPs unique to this single specimen (*C. montana* 50) led to an over-representation of SNPs with an *F*_*ST*_ of 1. *F*_*ST*_ was calculated with these individuals and species: two *C. angustifolia* (specimens 88, 143); ten *C. fissilis* (specimens 9, 19, 112, 130, 140, 211, 230, 254, 264, 292), seven *C. odorata* (specimens 10, 52, 162, 185, 202, 222, 277); and three *C. saltensis* (specimens 75, 102, 186). For the 215,799 biallelic SNPs with no missing information, the mean *F*_*ST*_ was 0.2, with a range of 0–1 (Fig. 4; light grey bars); the mean and range of *F*_*ST*_ for the stringently filtered subset of 119,020 biallelic SNPs was essentially identical (Fig. 4; bars outlined in black). We determined that 71,441 of the stringently-filtered SNPs appeared on 5693 gene targets with associated gene family and gene ontology information (Fig. 4; white portion of bars outlined in black). Of the stringently-filtered SNPs, 22,074 SNPs (or 5.6% of the detected variants) had an *F*_*ST*_ ≥ 0.5, and 9081 of the candidate SNPs (2.1%) had a *F*_*ST*_ of 1, indicating a high degree of fixation among species. This subset of high-quality SNPs provides a pool of candidate SNPs for that can be used for the identification of *Cedrela* wood specimens to species.

### Chloroplast genome assembly and variation

DNA sequencing of CEOD-NYBG generated 2.3 × 10^8^ paired sequences (46.7 Gb), and these were used unmodified for de novo chloroplast genome assembly using NOVOPlasty [34]. NOVOPlasty used 3.5 × 10^5^ reads (0.2%) to assemble three contigs, one with 140,933 bp and two that were 19,220 and 19,117 bp in length. Alignment to the *Azadirachta indica* A. Juss. chloroplast genome [35] revealed that the largest contig included two inverted repeat regions separated by the large single-copy region. The two remaining 19 Kbp contigs represented both orientations of the small single-copy region; we retained the small single-copy contig that matched the orientation of the *A. indica* chloroplast genome. Since we did not know the size of the putative missing sequences at the two contig junctions, contig boundaries were separated by 100 “N” bases. The final length of the reordered assembly was 160,153 bp, and GC content was 37.9%. By mapping the original paired sequences back to this reference, we determined that chloroplast DNA accounted for 1.4% of the mapped reads (6.3 × 10^6^), and the final depth of coverage of the assembly was estimated at nearly 4000X.

RAST [36] annotated 269 elements in the CEOD-NYBG draft chloroplast genome. Of the 269 elements, 184 (68.4%) were hypothetical proteins and 85 (31.6%) were genes that could be associated with function. Some of the elements that could be associated with function could be grouped into subsystem categories: twenty-one were associated with photosynthesis, five with RNA metabolism, four with respiration, two with protein metabolism, and one with carbohydrate metabolism.

**Fig. 5:**
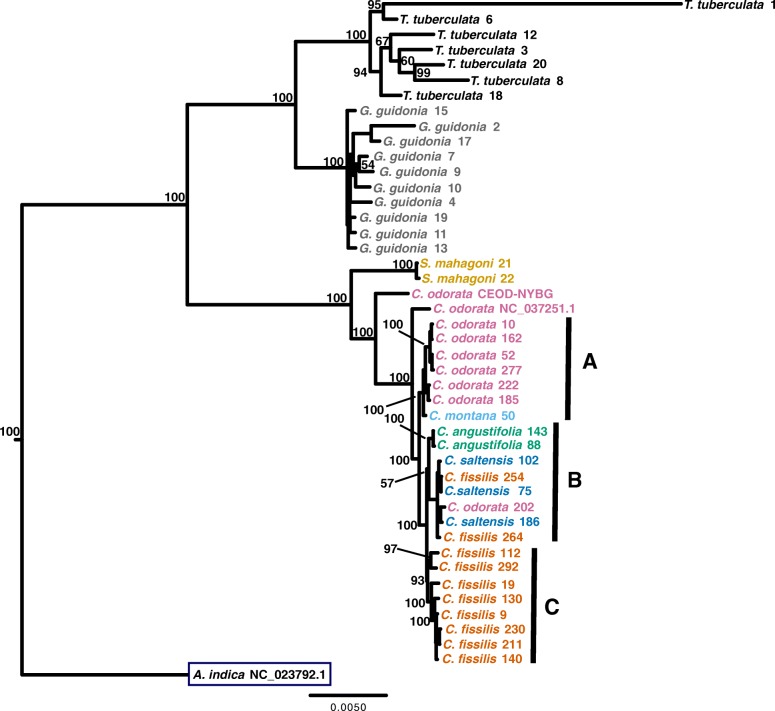
Maximum likelihood tree inferred from whole chloroplast genomes. Taxa are color coded by species. Numbers near branches correspond to a subset of bootstrap support values for 1000 bootstrap replicates (see Additional file 1: Figure S3)

Once assembled, we used the CEOD-NYBG chloroplast genome reference to guide chloroplast genome assembly for the additional specimens used for target capture. Coverage statistics showed that the number of reads with chloroplast identity averaged 4.3 × 10^5^ (range: 3.6 × 10^4^–3.8 × 10^6^) across all samples (Table 2). Chloroplast genome coverage averaged 92% (range: 61–100%), and the mean depth of coverage was 280X (range: 25 - 2399X). Following genome alignment with MAFFT ([37, 38]; see Methods), we used RAxML [39] to infer the maximum likelihood phylogeny of chloroplast genomes with 1000 bootstrap replicates (Fig. 5; see Additional file 1: Figure S3). This phylogeny included the CEOD-NYBG reference, chloroplast genomes we prepared by reference-guided assembly, and two publicly available chloroplast genomes from the Meliaceae: *A. indica* NC_023792.1 and *C. odorata* NC_037251.1 [35, 40]. Our alignment for all taxa contained 11,950 SNPs, 1695 of which were segregating within *Cedrela* taxa (~ 7.6 SNPs/Kbp). All genera resolved as monophyletic with strong bootstrap support, despite low-coverage assemblies for some *G. guidonia* and *T. tubderculata* (Table 2). While *Cedrela* taxa resolved as a single clade, *Cedrela* genomes were not monophyletic within species, with the exception of *C. angustifolia*. Most strikingly, the CEOD-NYBG reference specimen from Oaxaca, Mexico resolved as sister to all *Cedrela* in our analysis; a similar placement was also observed with the *C. odorata* NC_037251.1 [40] from Cuba (Fig. 1; see Additional file 1: Table S1). These geographically distant specimens possessed genetically divergent haplotypes relative to South American *C. odorata* that appeared to predate the divergence of chloroplast genomes currently residing in South American *Cedrela* species.

Within *Cedrela*, we designated three subclades to aid interpretation (Fig. 5). Subclade A contained only Central American *Cedrela* but also included *C. montana* 50 from Colombia (Figs. 1 and 5). Given that we included a single *C. montana* sample in this analysis, we don’t know whether this resolution is typical or anomalous for *C. montana*. The remaining *C. odorata* in Subclade A resolve into three strongly supported sister pairs (99–100% bootstrap support) that are congruent with geography: *C. odorata* 10 and 162 from Nicaragua, *C. odorata* 52 and 277 from Costa Rica, and *C. odorata* 222 and 185 from Panama.

Subclade A was sister to a South American lineage of two subclades, B (weakly supported; 57% bootstrap support) and C (strongly supported; 93% bootstrap support; Fig. 5; see Additional file 1: Figure S3). Subclade B contained representatives of four species from the same geographic region of Bolivia (Gran Chaco), with the exception of *C. odorata* 202 from Venezuela (Figs. 1 and 5). This clade also contained the only monophyletic *Cedrela* species in our study, *C. angustifolia*. The remaining taxa in this clade included a mixture of *C. fissilis* and *C. saltensis* specimens. Given the geographic proximity of these specimens and the weak differentiation of linages indicated by low bootstrap values, chloroplast haplotype variation likely reflects a combination of recent primary divergence, potential secondary contact and introgression/hybridization, and geographic structure. Subclade C also showed evidence of phylogenetic and geographic signal (Fig. 5). This clade was composed exclusively of geographically proximate *C. fissilis* samples from northern Bolivia and Southern Brazil (Fig. 1), with the exception of *C. fissilis* 112 from Ecuador.

## Discussion

The main objective of this study was to design hybridization capture probes that enriched targets from the genomes of *Cedrela* species with the ultimate goal of applying genome-scale resources to species identification and spatial origin assignment of wood specimens and population genomics studies. Although we don’t address technical issues associated with DNA extraction from wood and species identification, we provide a set of markers for species validation that can be tested by groups developing protocols for genotyping DNA from *Cedrela* wood [39]. Additionally, we present a framework to develop custom genetic markers to screen wood DNA of other tree species accompanying existing protocols for DNA extraction from wood [41–48].

### A transcriptome reference and genomic resources for *Cedrela*

Our first step required the assembly a draft transcriptome for *Cedrela odorata*, a member of the subfamily Cedreloideae, a group that includes many prized timber species (e.g., *Swietenia, Khaya, Entandophragma* [49]) that are the focus of agroforestry and breeding, as well as targets of illegal logging. Despite the economic importance of the mahogany family as a source of timber and medicine [50–53], few transcriptome and genome resources from the ~ 600 described species in this family exist. Transcriptomes have only been described for four species, *Azadirachta indica* [54], *Carapa guianensis* [55], *Toona sinensi* [56], and *Melia azedarach*; reference assemblies are publicly available for two of these transcriptomes (1KP project; https://sites.google.com/a/ualberta.ca/onekp/). The transcriptome assembly we present here includes over 52,000 transcript models totaling 47.8 Mbp, and 60.3% of transcripts had gene family and gene ontology information in public databases. Our transcriptome was made from a single tissue and developmental stage (mature leaflets), and it contains 65–70% as many transcripts and assembled transcript bases as fully-characterized tree genomes like *Populus trichocarpa* (73,014 transcripts, 69.2 Mbp; 41,479 protein-coding genes; [57]) or *Quercus lobata* (83,644 transcripts, 72.5 Mbp; 61,773 protein-coding genes; [58]). A larger proportion of *P. trichocarpa* genes are associated with gene families (91%) and gene ontology terms (78%) than our gene models for *C. odorata*; however, we captured a similar number of multispecies gene families (7823 in *P. trichocarpa* [48]; 6043 in our *C. odorata*). Additional effort in transcript sampling (tissues and developmental stages) and annotation is needed to fully describe the transcriptomic and metabolic complexity of *C. odorata*.

We identified 10,001 low-copy gene targets from *C. odorata*. Our strategy enriched gene targets in the 10th – 30th percentile with regard to exon or transcript number, therefore avoids genes with a higher degree of duplication across the genome of *C. odorata,* which is presumably tetraploid [1, 10, 11]. Target enrichment of 10,001 gene targets (using 19,740 hybridization probes) showed a high degree of success, as enrichment was demonstrated for all but 206 targets (2% of total). While we demonstrated that these hybridization probes were sufficient for sampling the genomes of 43 specimens, these probes could be further optimized and expanded. Future designs should exclude the failed gene targets, as well as gene targets that enriched a disproportionately large amount of sequence. It’s possible that these targets represent multi-copy genes in *Cedrela*. Removing these gene targets would improve the uniformity of sampling across targets, increase the average depth for lower-depth targets, and increase the number of reliably enriched genes across the specimens included in the diversity panel.

### Target enrichment of putative homologues from the Meliaceae

We used *C. odorata-*derived hybridization probes to enrich target genes from a diversity panel of 43 specimens representing four genera from the mahogany family (*Cedrela, Guarea, Swietenia,* and *Trichilia*). By normalizing the sequence read input across species to 1 million organelle-depleted DNA sequences, our results showed that the depth of coverage across *Cedrela* targets converged on the same mean, regardless of species, sample size, or individual sequence yield (Fig. 5; Table 2). This is consistent with previous studies employing similar techniques that found sufficient enrichment of targets at the generic level in other plants like *Pinus* [59, 60], *Sabal* [61], and *Asclepias* [62]. Consistent mean sampling depth across *Cedrela* species demonstrates that this selection of hybridization probes, designed from *C. odorata* gene models, shows minimal bias for sampling homologous gene targets across the five *Cedrela* species included in our study. This is relevant for the three CITES-listed *Cedrela* species, as it demonstrates that future efforts to develop genomic information for these species with hybridization enrichment should show a high degree of success. Similarly, while our experiment included 5 of 18 named *Cedrela* species, the high transferability of these probes across sampled species makes it likely that they will work with equal efficiency for the 13 *Cedrela* species not included our study.

We imposed a stringent depth of coverage threshold of 10X across all specimens for each species to determine the number of target genes that could be reliably sampled with our probe set (range for *Cedrela* species: 3093 – 8794). Our rationale for adopting such stringent evaluation criteria is that downstream applications in wood-based DNA identification require targets to have a high expectation for success and reproducibility, leading to likelihoods of individual or spatial assignment when evidence is evaluated in legal proceedings [63]. Target enrichment ‘drop-out’ can occur due to methodological factors (e.g., sampling probability across a large number of targets), as well as biological, locus-specific features that make sampling less reliable (e.g., position of probes relative to indels or introns; base composition) or preclude sampling altogether (e.g., locus deletion; copy number variation) [18, 19]. By excluding genes that show variable enrichment success, we can avoid genes that show sampling bias due to biological phenomena.

We identified over 2 × 10^5^ biallelic SNPs that show no missing information across our sample of *Cedrela* species and individuals. Moreover, this sample includes 9000 high-quality SNPs that show strong differentiation between one or more *Cedrela* species (e.g., *F*_*ST*_ of 1). This subset of high-quality SNPs should be useful for classifying *Cedrela* wood to species. An important caveat of this result is that the ‘global’ *F*_*ST*_ was calculated from small sample sizes (most notably *C. angustifolia* (*n* = 2) and *C. saltensis* (*n* = 3); *C. montana* (*n* = 1) was excluded for this reason). Small sample sizes can inflate the number of high *F*_*ST*_ SNPs by failing to accurately account for the variation within species. Additionally, high *F*_*ST*_ SNPs will disproportionately represent divergence events separating the most genetically divergent lineage. Future refinement of ‘species-specific’ SNPs will require a larger sample size, and analysis methods that partition variation for the identification of species-diagnostic SNPs, such as pairwise *F*_*ST*_ or machine learning algorithms. This will be particularly important for the task of separating species like *C. odorata* and *C. fissilis*, which have large overlapping geographic ranges, show a close phylogenetic affinity [1, 8], and are CITES-listed species [2].

By including samples from the subfamilies Celedroideae (*Swietenia*) and Melioideae (*Guarea, Trichilia*), we were able to evaluate the potential for transferring *Cedrela*-derived probes to similar genetic studies across the Meliaceae. While the hybridization probes derived from *C. odorata* showed considerable bias for *Cedrela*, enrichment appears feasible at the level of subfamily [62], as we were able to reliably enrich 3957 genes from *S. mahagoni*, the sole representative from the Celedroideae (Fig. 2). Operationally, this means that a large number of gene targets should be cross-compatible with close (e.g., Asian *Toona*) and distant relatives from other genera (e.g., American and Neotropical *Swietenia* species, all of which are CITES listed). Members of the Melioideae showed considerably lower target enrichment success (Fig. 3), with 774 and 565 enriched genes from *G. guidonia* and *T. tuberculata*, respectively (Fig. 2). While these representatives showed poor enrichment relative to *Cedrela* (Table 2), this number of genes is comparable to the yield of more focused target enrichment strategies used for population and evolutionary studies of Neotropical trees (e.g., 264 nuclear loci in *Inga* [64]), and it far exceeds the currently available genetic information from *G. guidonia* and *T. tuberculata* in the National Center for Biotechnology Information (NCBI). Recent studies have developed target capture probes for a broader phylogenetic scope in plants [65] and animals [66] by selected ultra-conserved genes across taxa for probe design; similar approaches could be applied to the Meliaceae, after the release of additional transcriptomes.

### Chloroplast variation tracks geography among *C. odorata* specimens

Studies focusing on chloroplast genome variation among species in *Cedrela* [8, 67], and more broadly for the mahogany family [49], have provided a useful taxonomic framework for this study. We add an additional de novo chloroplast genome reference for *C. odorata* to a growing collection of Meliaceae plastid sequences. Our chloroplast genome was adequate for the reference guided assembly for 43 Meliaceae specimens, but chloroplast genome assembly fragmentation increased with increasing phylogenetic distance from *C. odorata* [49]. The reduction in completeness of the chloroplast genomes for *G. guidonia* and *T. tuberculata* led to long branch lengths (Fig. 5) that were likely due to assembly error, rather than phylogenetic divergence among specimens originating in Panama. By selecting a more genetically proximal assembly reference from subfamily Melioideae (e.g., *A. indica*), improved assemblies for these taxa should be possible with the sequences presented here.

Our inferred chloroplast genome phylogeny from this limited sample of *Cedrela* shows a general lack of species monophyly, as only *C. angustifolia* resolved as monophyletic in the five tested *Cedrela* species. This result is consistent with the known taxonomic complexity in this genus from hybridization [68], presumably paraphyletic taxa [9, 13], and proposed the existence of ‘cryptic’ species [8, 13]. In our sample, chloroplast genomes appear to be more informative for geographic origin than for taxonomic classification. Cavers et al. (2013) reported a similar trend in *C. odorata*, which showed spatial genetic structure among chloroplast haplotypes (two chloroplast genes, three chloroplast microsatellites) that reflected latitudinal position. This blurring of taxonomic and geographic signal from the chloroplast has been observed in other angiosperm taxa due to introgression and incomplete lineage sorting, with examples such as *Acer* [69], *Betula* [70], *Quercus* [71], and others [72, 73]. While more testing is required to confirm the utility of whole chloroplast genomes as a geographic marker for *Cedrela*, whole or partial chloroplast genomes show promise for broad-scale geographic source identification for wood specimens.

## Conclusions

We present a set of hybridization probes for target capture of genomic libraries from *Cedrela* species. The probe set is based on gene models selected from a de novo transcriptome assembly from *C. odorata,* and selected to represent low-copy genes in the subgenomes of this tetraploid species. The probe set can be successfully applied across the genus *Cedrela* and subfamily Cedreloideae, and it may show limited success with more divergent relatives in the Meliaceae (as demonstrated by obtaining hundreds of genes at reliable depth for distant species *G. guidonia* and *T. tuberculata*). In addition to this probe set, we provide draft chloroplast genome assemblies for 43 specimens across eight Meliaceae species, including *C. odorata*. By comparing SNP frequencies between species, we provide a set of candidate SNPs for species discrimination of *C. angustifolia*, *C. fissilis*, *C. odorata*, and *C. saltensis*, and we provide preliminary support for the chloroplast genome as a marker of geography for *Cedrela,* and *C. odorata* in particular. These genomic resources for *Cedrela* and the Meliaceae will enable further detailed genetic study of *Cedrela*, a genus that contains three species of conservation concern and many species that yet to be assessed for conservation status. These resources from target capture should provide a level of resolution that can support the development of genetic screening tools for timber from *Cedrela,* aiding in the enforcement of CITES regulations for trade in this historically over-exploited plant group.

## Methods

### DNA preparation and sequencing of the reference specimen

Fresh leaf tissue was collected from *Cedrela odorata* at NYBG (Fig. 1; see Additional file 1: Table S1; NYBG Accession 683/89; Bronx, NY, USA; Oregon State University Herbarium voucher OSC-V-258305; referred to as “CEOD-NYBG”), and frozen on dry ice for total RNA and DNA extraction (Norgen Plant RNA/DNA Purification Kit, Norgen Biotek, Thorold, Ontario, CAN). RNA and DNA were quantified by fluorometry (Qubit, Thermo Fisher Scientific, Waltham, MA, USA), and RNA was treated with DNase using the TURBO DNA-free Kit (Life Technologies, Thermo Fisher Scientific, Carlsbad, CA, USA). RNA quality and size distribution were assessed using the Agilent 2100 Bioanalyzer (Agilent Technologies, Santa Clara, CA, USA). To construct a poly(A)-selected RNA-seq library, we used the WaferGen Bio-systems Apollo 324 NGS Library Prep System and 500 ng of total RNA (WaferGen Bio-systems, Fremont, CA, USA). To construct the DNA library, 500 ng of total genomic DNA was sheared to a modal size of 130 bp using sonication (30 cycles; 30 s on + 30 s off; Diagenode BioRuptor, Denville, NJ, USA), and adapted an Illumina genomic library using the NEBNext Ultra II Kit (New England Biolabs Inc., Ipswich, MA, USA). The mRNA and DNA libraries from CEOD-NYBG were uniquely indexed, pooled to equal mass representation, and sequenced using one lane of 100 bp paired-end sequencing on an Illumina HiSeq 3000 (Center for Genome Research and Biocomputing [CGRB], Oregon State University, Corvallis, OR, USA).

### DNA preparation and sequencing of the diversity panel

Leaf tissue was obtained from three sources to assess the utility and transferability of our genomic resources (see Additional file 1: Table S1; specimens collectively referred to as the “diversity panel”): herbarium specimen fragments at the Missouri Botanical Garden Herbarium (MO; St. Louis, MO, USA), the living collection at Fairchild Tropical Botanic Garden (FTBG; Coral Gables, FL, USA), and five locations in Panama. Our collections from MO included twenty-three specimens from five *Cedrela* species: *C. angustifolia* DC. (*n* = 2), *C. fissilis* Vell. (*n* = 10), *C. montana* Moritz ex Turcz. (*n* = 1), *C. odorata* L. (*n* = 8), and *C. saltensis* M. A. Zapater & del Castillo (*n* = 3). Figure 1 shows the origin of *Cedrela* specimens. We extracted total genomic DNA from MO specimens with the FastDNA Kit (MP Biomedicals, Santa Ana, CA, USA). For genera outside of *Cedrela*, we obtained: *Sweitenia mahagoni* (L.) Jacq. (*n* = 2; FTBG), *Trichilia tuberculata* C. DC. (*n* = 7; Panama), and *Guarea guidonia* (L.) Sleumer (*n* = 10; Panama). Total genomic DNA was extracted from FTBG *S. mahagoni* specimens with the DNeasy plant mini kit (Qiagen, Hilden, Germany). Panama specimens were collected fresh, dried, and stored frozen until DNA extraction with the FastDNA Kit. Genomic DNA from the MO, FTBG, and Panama specimens were quantified by fluorometry, sheared to a modal size of 130 bp, and converted to Illumina libraries as described above. We pooled DNA libraries to equal molar representation for hybridization capture and targeted enrichment (see ‘Target Capture’ section). After target capture, libraries were pooled and sequenced using one lane of 100 bp paired-end sequencing on an Illumina HiSeq 4000 (Genomics & Cell Characterization Core Facility at University of Oregon, Eugene, OR, USA). To gain additional sequences for the *Cedrela* specimens, the original (unenriched) multiplex and target-enriched multiplex were also each sequenced in separate sequencing reactions, at a proportional representation of approximately ¼ of a HiSeq lane (multiplexing performed with samples from a separate study). These libraries were sequenced using 100 bp paired-end sequencing on the Illumina HiSeq 3000 at the Oregon State CGRB.

### Draft transcriptome assembly

We assembled the transcriptome of the CEOD-NYBG reference specimen following the de novo transcriptome assembly pipeline [22, 74] developed by the National Center for Genome Resources (Santa Fe, NM, USA). Except where specified, default settings were used for all bioinformatics software programs. Reads from the mRNA-seq library were trimmed using Trimmomatic v. 0.36 [75] with a 4 bp window, sequences that showed an average quality below a phred score of 15 (ASCI_BASE 33) were cropped, adapter sequences were removed, N bases (or sequencing quality below a threshold of 3) were cropped from the leading strand and trailing strand, and sequences less than 36 bp in length were filtered out. We used FLASH v. 1.2.4 [21] to combine overlapping forward and reverse reads into super reads. Super reads and unextended, paired reads were assembled de novo as unitigs with an ABySS v. 1.3.7-maxk128 [23, 76] k-mer sweep (*K* = 55, 67, 73, and 89). ABySS unitigs were concatenated and filtered with CD-HIT v. 4.5.5 [77, 78] to reduce redundancy of unitigs with 98% identity. Filtered unitigs were then assembled into contigs with CAP3 v. 20071015 [79] and scaffolds were generated with the ABySS scaffold function of run-bpa.pl with a kmer length of 77 bp [74]. We reduced the number of gaps in the transcriptome with GapCloser v. 1.12 for SOAP de novo [80] by setting the maximum read length to 187 bp for FLASH-extended super reads, 101 bp for unextended paired reads, and an average insert size of 100. GapCloser was repeated until assemblies no longer improved. CD-HIT was used again to reduce redundancies among transcript models. A final filtering step removed transcript models with less than 200 bp. To generate statistics on our final assembly, we used TransRate v. 1.0.3 for Linux [81]. We obtained gene family and ontology information for our draft transcriptome with TRAPID [25] and the PLAZA 2.5 data source [26] using *Populus trichocarpa* as the similarity reference [57].

### Hybridization probe design

Using 10,001 transcript models (or “gene targets”) representing low-copy genomic regions in the CEOD-NYBG genome, we designed probes for hybridization capture and target enrichment (target capture). To identify low-copy gene targets, we masked repeats and low-complexity regions of the draft transcriptome with RepeatMasker v. 3.3.0 [82, 83] using *Arabidopsis thaliana* as the repeat reference. Then, we cropped 101 bp reads from the CEOD-NYBG genomic DNA library to produce 50 bp ‘subreads’ (Trimmomatic v. 0.36 [75]), which were then locally aligned to the transcriptome with the bbmap.sh tool of BBTools v. 36.14 [29]. Subreads were mapped using a threshold of 95% identity, and mapping conflicts were resolved by retaining multiple high-quality mapping locations. The bbmap.sh “covstats” parameter was used to obtain coverage estimates for all transcript models. Using the covstats data, we estimated the number of mapped genomic reads per 1 Kbp of transcript length (RPK), and we sorted the transcript models by RPK in ascending rank order. Transcript models ranked from 5000 to 15,000 (out of 52,181 total) were chosen as gene targets for target capture. These gene targets were used to design 19,740 custom 100 bp biotinylated RNA hybridization probes (MYbaits™, Arbor Biosciences, Ann Arbor, MI, USA), with two RNA probes per gene target tiled end-to-end beginning at the 5′ end of the gene target.

### Target capture

Target capture with MYbaits™ was performed on the genomic DNA libraries prepared from the diversity panel of specimens as directed by the manufacturer. Prior to capture, libraries were pooled in equimolar 24-plex pools for the *Cedrela* specimens, and as equimolar 19-plex pools for *S. mahagoni*, *G. guidonia*, and *T. tuberculata* genomic libraries. For the 19-plex pools, we used a lower hybridization temperature of 58 °C (as opposed to 65 °C) to accommodate more divergent gene targets. Post-capture enriched targets were amplified using the KAPA HiFi HotStart ReadyMix PCR Kit v. 5.13 (KAPA Biosystems, Boston, MA, USA) and NEXTFlex Primers (Bioo Scientific Corporation, Austin, TX, USA), following the manufacturer’s protocol. We pooled the amplified, captured *Cedrela* and *S. mahagoni*/*G. guidonia*/*T. tuberculata* libraries at 1:1 M ratios and then added 1/8 mass proportion of unenriched genomic library from eight specimens (*T. tuberculata* 1, 3, 18; *G. guidonia* 2, 7, 11; *S. mahagoni* 21, 22) to increase chloroplast genome coverage for these taxa. This pool was sequenced to obtain paired-end 100 bp sequences using one lane of the Illumina HiSeq 4000 (Genomics and Cell Characterization Core Facility, University of Oregon, Eugene, OR, USA).

We assessed sequence yield and on-target yield by mapping captured DNA sequence reads from the specimens back to the gene targets from CEOD-NYBG at 90% identity in local alignment mode with bbmap.sh. At this point, we determined that specimen *C. odorata* 287 yielded fewer than 10^6^ reads (Table 2). We included it for total sequence yield calculations, but excluded it from all other analyses and calculations. On-target yield calculations were based on the covstats parameter and summarized by individual and species using R scripts [84]. Individual on-target yield was calculated by estimating the sum of sequenced reads that mapped to each gene target (‘plus’ and ‘minus’ strands). We calculated depth by multiplying the sequence read length (101 bp) by the number of sequenced reads mapping to each gene target (‘plus’ and ‘minus’ strands) divided by the covered target length (‘covered bases’). The unpaired Student’s *t* test was used to determine whether mean on-target sequence yield was higher for *Cedrela* specimens versus the other Meliaceae specimens. These data, detailed explanations of our analysis in R, and other resources from this study are available through the Oregon State University Scholars Archive [24]. All R packages used for analysis are listed in Additional file 2: Table S2.

On-target depth per gene target estimates were used to identify ‘reliably enriched regions’ that exceeded a depth of 10 for every specimen within a species (*C. angustifolia, n =* 2*; C. fissilis*, *n =* 10; *C. montana, n =* 1*; C. odorata*, *n =* 7; *C. saltensis, n =* 3*; G. guidonia, n =* 10; *S. mahagoni*, *n =* 2; *T. tuberculata, n =* 7). TRAPID gene association information was used to identify the proportion of gene families that could be inferred from reliably enriched gene targets for each species.

To estimate sequence (SNP) variation from *Cedrela* specimens, we mapped the sequenced reads from each individual using the 10,001 gene targets from CEOD-NYBG as a reference with BWA-MEM [85, 86]. Once reads were aligned, we used SAMtools [30] to convert and sort the alignment files, and the Genome Analysis Tool Kit (GATK) v. 3.7 [31] to define and realign insertions and deletions. SAMtools mpileup was used to generate a vcf [24], and VCFtools was employed to perform variant filtering [33]. Using these programs, we applied stringent filters to identify a ‘high-confidence’ set of SNPs to differentiate *Cedrela* species. First, we removed SNPs that showed missing information for any of the individuals, and then removed SNPs that showed minor allele frequency less than 5%. Second, we used the ‘quality metric’ of VCFtools (defined as -10Log_10_[*probability of incorrect SNP call*]) to identify and select SNPs showing a high probability of accuracy, defined here as ‘quality *>* 500’. After filtering, we used VCFtools to calculate Weir and Cockerham’s *F*_*ST*_ [32] for SNPs on a per marker basis, using *Cedrela* species as ‘populations’ in these calculations.

### Target capture efficiency

We assessed target capture efficiency of the CEOD-NYBG*–*derived hybridization probes on other *Cedrela* species and Meliaceae genera by normalizing read count, thus removing the effect of taxon-specific enrichment bias. To do this, we removed sequence reads that showed 90% chloroplast identity with our CEOD-NYBG draft chloroplast genome (bbmap.sh; see next section), and subsampled 10^6^ paired-end sequence reads from each taxon (reformat.sh; BBTools). We mapped subsampled reads by individual to the 10,001 gene targets in local alignment mode with bbmap.sh at 90% identity. We used covstats for each specimen allowing us to estimate depth per gene target, individual mean depth for each specimen, and species mean depth for the eight species groups. We calculated depth as described above, and used an unpaired Student’s *t* test to determine whether mean on-target depth was higher for *Cedrela* specimens than for the other Meliaceae specimens.

### Draft chloroplast genome assemblies

We generated a seeded, overlap-extension chloroplast genome assembly for CEOD-NYBG using genomic DNA sequence reads and NOVOPlasty v. 2.6.2 [34]. *C. odorata rbcL* (AY128220.1) [49] was used as the seed sequence, and NOVOPlasty parameters were set to default except: insert size (300 bp), auto-detect insert size (on), read length (101 bp), and k-mer length (23). We aligned the resulting chloroplast contigs to the draft chloroplast genome of *A. indica* (NC_023792.1) [35] via the LASTZ-based MULAN aligner [87]. Gene features were annotated using RAST [36].

For each specimen, we concatenated sequence files from all available experiments (e.g., target capture experiments; genome skimming), and mapped reads from each specimen to the CEOD-NYBG chloroplast reference using bbmap.sh in local alignment mode, a 90% identity threshold, and randomly mapping multi-mapped reads. The resulting binary alignment files (BAM) were loaded into Geneious v. 7 [88] with the CEOD-NYBG chloroplast reference. For each specimen, a consensus sequence was generated where bases were coded as ‘N’ if coverage was less than 2X. Sequences were exported as separate FASTA files for subsequent alignment and phylogenetic analysis [24].

We used BBTools covstats to estimate chloroplast abundance in total DNA by calculating the sum of sequenced reads with chloroplast identity for each specimen. Depth of coverage for the chloroplast genomes from the diversity panel was estimated as described above. We also estimated the percent of the reference covered by at least one mapping read using covstats. After compiling draft chloroplast genomes, we used a custom python script to estimate the abundance of ambiguous bases (‘N’) for each genome [24].

### Phylogenetic analysis of chloroplast genomes

Compiled FASTA files containing draft chloroplast genomes from the diversity panel were combined with the CEOD-NYBG draft genome and two publicly available Meliaceae chloroplast reference genomes (*A. indica* NC_023792.1 and *C. odorata* NC_037251.1 [35, 40]), and then aligned using the “FFT-NS-2” alignment method implemented in MAFFT [37] using default parameters. We used this alignment to determine the number of SNPs detected among all taxa and among only *Cedrela* taxa with Mesquite v. 3.31 [build 859] [89]. Phylogenetic relationships for *Cedrela* and other Meliaceae representatives were inferred with maximum likelihood and 1000 bootstrap replicates using with RAxML v. 8.2.10 [39] via the CIPRES online server [90]. For this analysis, the default secondary structure substitution model was selected (16-state general time reversible [GTR]), and *A. indica* was designated as the outgroup. All other parameters were default. A bootstrap majority rule consensus tree was generated with Mesquite with the required frequency of clades set to 0.5, and bootstrap support values (converted to percent) were superimposed over the RAxML best tree result obtained from CIPRES. Output trees were viewed and edited in FigTree v. 1.4.3 [91].

## Additional files

Additional file 1: **Table S1.** Specimen source and collection information. **Figure S1.** Distribution of Log_2_(Mapped Reads) for the gene models. **Figure S2.** Alternative view of main text Fig. 3. **Figure S3.** Bootstrap consensus maximum likelihood species tree inferred from whole chloroplast genomes. (DOCX 509 kb)

Additional file 2: Table S2. List of R packages used with version and citation. (DOCX 20 kb) **Finch, K. N. (2018).** Dataset for genomic resources for the neotropical tree genus Cedrela (Meliaceae) and its relatives [Data set]. Oregon State University. https://doi.org/10.7267/NV935820Q. Readers will find: the assembled transcriptome reference, hybridization capture probe sequences, the chloroplast genome reference for CEOD-NYBG, chloroplast genome sequences for each of the 43 specimens screened in our diversity panel (as separate files and as a combined file, aligned and unaligned), the VCF file containing SNPs for species and origin prediction for *Cedrela*, data sets to replicate our statistical analysis using R. (158 MB)

## Abbreviations

‘N’ bases: bases at lower than 2X coverage indicating an ambiguous base call
BAM: binary alignment files with file extension .bam
BOL: Bolivia
bp: base pair/s
BRA: Brazil
CEOD-NYBG: *Cedrela odorata* specimen obtained from the New York Botanical Garden and used for transcriptome reference assembly and target capture probe design (See Additional file 1: Table S1).
CITES: Convention on International Trade in Endangered Species of Wild Fauna and Flora
COL: Colombia
covstats: bbmap.sh flag used to generate coverage statistics after read mapping
CP: chloroplast
cpDNA: chloroplast DNA
diversity panel: 43 specimens used for hybridization capture and target enrichment
ECU: Ecuador
FAJ: Dr. F. Andrew Jones
FTBG: Fairchild Tropical Botanic Garden
Gbp: gigabase pairs
gene targets: 10,001 transcript models selected for hybridization probe design
GTR: general time reversible
Kbp: kilobase pairs
KNF: Kristen N. Finch
MAF: minor allele frequency
Mbp: megabase pairs
MEX: Mexico
MO: Missouri Botanical Garden Herbarium
NCBI: National Center for Biotechnology Information
NCGR: National Center for Genome Resources
NIC: Nicaragua
NYBG: New York Botanical Garden
ORF/s: open reading frame/s
PAN: Panama
PER: Peru
RCC: Dr. Richard C. Cronn
RPK: reads per Kbp
SNP/s: single nucleotide polymorphism/s
Target capture: our abbreviation for hybridization capture target enrichment and short read sequencing
VCF: variant call format file with file extension .vcf
VEN: Venezuela

## References

[1] Pennington TD, Muellner AN (2010). A monograph of *Cedrela* (Meliaceae).

[2] UNEP-WCMC (2015). Overview of CITES Appendix III listings.

[3] Muellner A, Schaefer H, Lahaye R (2011). Evaluation of candidate DNA barcoding loci for economically important timber species of the mahogany family (Meliaceae). Mol Ecol Resour.

[4] Gasson P (2011). How precise can wood identification be? Wood anatomy’s role in support of the legal timber trade, especially CITES. IAWA J.

[5] Dormontt EE, Boner M, Braun B, Breulmann G, Degen B, Espinoza E (2015). Forensic timber identification: It’s time to integrate disciplines to combat illegal logging. Biol Conserv.

[6] Ogden R. Unlocking the potential of genomic technologies for wildlife forensics. Mol Ecol Resour. 2011;11:109–16. 10.1111/j.1755-0998.2010.02954.x 21429167

[7] Ogden R, Linacre A (2015). Wildlife forensic science: a review of genetic geographic origin assignment. Forensic Sci Int Genet.

[8] Cavers S, Telford A, Arenal Cruz F, Pérez Castañeda AJ, Valencia R, Navarro C (2013). Cryptic species and phylogeographical structure in the tree *Cedrela odorata* L. throughout the Neotropics. J Biogeogr.

[9] Muellner AN, Pennington TD, Koecke AV, Renner SS (2010). Biogeography of *Cedrela* (Meliaceae, Sapindales) in central and South America. Am J Bot.

[10] Styles BT, Khosla PK, Burley J, Styles BT (1976). Cytology and reproductive biology of Meliaceae. Tropical trees: variation, breeding and conservation.

[11] Styles BT, Vosa CG (1971). Chromosome numbers in the Meliaceae. Taxon.

[12] Wendel JF, Schnabel A, Seelanan T (1995). Bidirectional interlocus concerted evolution following allopolyploid speciation in cotton (*Gossypium*). P Nat Acad Sci USA.

[13] Garcia MG, Silva RS, Carniello MA, Veldman JW, Rossi AAB, de Oliveira LO (2011). Molecular evidence of cryptic speciation, historical range expansion, and recent intraspecific hybridization in the Neotropical seasonal forest tree *Cedrela fissilis* (Meliaceae). Mol Phylogenet Evol.

[14] Mangaravite É, Vinson CC, Rody HV, Garcia MG, Carniello MA, Silva RS (2016). Contemporary patterns of genetic diversity of *Cedrela fissilis* offer insight into the shaping of seasonal forests in eastern South America. Am J Bot.

[15] Cavers S, Navarro C, Lowe AJ (2003). Chloroplast DNA phylogeography reveals colonization history of a Neotropical tree, *Cedrela odorata* L., in Mesoamerica. Mol Ecol.

[16] Cavers S, Navarro C, Lowe AJ (2003). A combination of molecular markers identifies evolutionarily significant units in *Cedrela odorata* L.(Meliaceae) in Costa Rica. Conserv Genet.

[17] Koecke AV, Muellner-Riehl AN, Pennington TD, Schorr G, Schnitzler J (2013). Niche evolution through time and across continents: the story of Neotropical *Cedrela* (Meliaceae). Am J Bot.

[18] Cronn R, Knaus BJ, Liston A, Maughan PJ, Parks M, Syring JV (2012). Targeted enrichment strategies for next-generation plant biology. Am J Bot.

[19] Heyduk K, Stephens JD, Faircloth BC, Glenn TC. Targeted DNA region re-sequencing. In: Aransay AM, JLL T, editors. Field Guidelines for Genetic Experimental Designs in High-Throughput Sequencing. Switzerland: Springer International Publishing; 2016. p. 43–68.

[20] Gnirke A, Melnikov A, Maguire J, Rogov P, LeProust EM, Brockman W (2009). Solution hybrid selection with ultra-long oligonucleotides for massively parallel targeted sequencing. Nat Biotechnol.

[21] Magoč T, Salzberg SL (2011). FLASH: fast length adjustment of short reads to improve genome assemblies. Bioinformatics.

[22] Keeling PJ, Burki F, Wilcox HM, Allam B, Allen EE, Amaral-Zettler LA (2014). The marine microbial eukaryote transcriptome sequencing project (MMETSP): illuminating the functional diversity of eukaryotic life in the oceans through transcriptome sequencing. PLoS Biol.

[23] Simpson JT, Wong K, Jackman SD, Schein JE, Jones SJ, Birol I. ABySS: a parallel assembler for short read sequence data. Genome Res. 2009;19:1–6. 10.1101/gr.089532.108 PMC2694472 19251739

[24] Finch KN. Dataset for genomic resources for the neotropical tree genus *Cedrela* (Meliaceae) and its relatives: Oregon State University, Oregon, USA; 2018. https://ir.library.oregonstate.edu/concern/datasets/nv935820q 10.1186/s12864-018-5382-6 PMC6339301 30658593

[25] Van Bel M, Proost S, Van Neste C, Deforce D, Van de Peer Y, Vandepoele K (2013). TRAPID: an efficient online tool for the functional and comparative analysis of de novo RNA-Seq transcriptomes. Genome Biol.

[26] Van Bel M, Proost S, Wischnitzki E, Movahedi S, Scheerlinck C, Van de Peer Y (2012). Dissecting plant genomes with the PLAZA comparative genomics platform. Plant Physiol.

[27] Lurin C, Andrés C, Aubourg S, Bellaoui M, Bitton F, Bruyère C (2004). Genome-wide analysis of *Arabidopsis* pentatricopeptide repeat proteins reveals their essential role in organelle biogenesis. Plant Cell.

[28] Apweiler R, Attwood TK, Bairoch A, Bateman A, Birney E, Biswas M (2001). The InterPro database, an integrated documentation resource for protein families, domains and functional sites. Nucleic Acids Res.

[29] BBTools. DOE Joint Genome Institute. https://jgi.doe.gov/data-and-tools/bbtools/. Accessed 27 Nov 2018.

[30] Li H, Handsaker B, Wysoker A, Fennell T, Ruan J, Homer N (2009). The sequence alignment/map format and SAMtools. Bioinformatics.

[31] McKenna A, Hanna M, Banks E, Sivachenko A, Cibulskis K, Kernytsky A, et al. The Genome Analysis Toolkit: a MapReduce framework for analyzing next-generation DNA sequencing data. Genome Res. 2010;20:1–8. 10.1101/gr.107524.110 PMC2928508 20644199

[32] Weir BS, Cockerham CC (1984). Estimating F-statistics for the analysis of population structure. Evolution.

[33] Danecek P, Auton A, Abecasis G, Albers CA, Banks E, DePristo MA (2011). The variant call format and VCFtools. Bioinformatics.

[34] Dierckxsens N, Mardulyn P, Smits G (2017). NOVOPlasty: *de novo* assembly of organelle genomes from whole genome data. Nucleic Acids Res.

[35] Kuravadi NA, Yenagi V, Rangiah K, Mahesh HB, Rajamani A, Shirke MD (2015). Comprehensive analyses of genomes, transcriptomes and metabolites of neem tree. PeerJ.

[36] Aziz RK, Bartels D, Best AA, DeJongh M, Disz T, Edwards RA (2008). The RAST server: rapid annotations using subsystems technology. BMC Genomics.

[37] Kazutaka K, Rozewicki J, Yamada KD; MAFFT online service: multiple sequence alignment, interactive sequence choice and visualization, Brief Bioinfor. 2017;bbx108:1-7. 10.1093/bib/bbx108 PMC6781576 28968734

[38] Katoh K, Standley DM (2013). MAFFT multiple sequence alignment software version 7: improvements in performance and usability. Mol Biol Evol.

[39] Stamatakis A (2014). RAxML version 8: a tool for phylogenetic analysis and post-analysis of large phylogenies. Bioinformatics.

[40] Mader M, Pakull B, Blanc-Jolivet C, Paulini-Drewes M, Bouda ZH-N, Degen B (2018). Complete chloroplast genome sequences of four Meliaceae species and comparative analyses. Int J Mol Sci.

[41] Asif MJ, Cannon CH (2005). DNA extraction from processed wood: a case study for the identification of an endangered timber species (*Gonystylus bancanus*). Plant Mol Biol Rep.

[42] Jiao L, Yin Y, Xiao F, Sun Q, Song K, Jiang X (2012). Comparative analysis of two DNA extraction protocols from fresh and dried wood of *Cunninghamia lanceolata* (Taxodiaceae). IAWA J.

[43] Tnah LH, Lee SL, Ng KKS, Bhassu S, Othman RY. DNA extraction from dry wood of *Neobalanocarpus heimii* (Dipterocarpaceae) for forensic DNA profiling and timber tracking. Wood Sci Technol. 2012;46:813–25.

[44] Dumolin S, Demesure B, Petit RJ (1995). Inheritance of chloroplast and mitochondrial genomes in pedunculate oak investigated with an efficient PCR method. Theor Appl Genet.

[45] Dumolin-Lapègue S, Pemonge M-H, Gielly L, Taberlet P, Petit RJ (1999). Amplification of oak DNA from ancient and modern wood. Mol Ecol.

[46] Deguilloux M, Pemonge M, Petit R (2002). Novel perspectives in wood certification and forensics: dry wood as a source of DNA. Pro Roy Soc Lond B Bio.

[47] Ogden R, McGough HN, Cowan RS, Chua L, Groves M, McEwing R (2008). SNP-based method for the genetic identification of Ramin *Gonystylus* spp. timber and products: applied research meeting CITES enforcement needs. Endanger Species Res.

[48] Lowe AJ, Wong K-N, Tiong Y-S, Iyerh S, Chew F-T (2009). A DNA method to verify the integrity of timber supply chains; confirming the legal sourcing of merbau timber from logging concession to sawmill. Silvae Genet.

[49] Muellner AN, Samuel R, Johnson SA, Cheek M, Pennington TD, Chase MW (2003). Molecular phylogenetics of Meliaceae (Sapindales) based on nuclear and plastid DNA sequences. Am J Bot.

[50] Lemes MR, Gribel R, Proctor J, Grattapaglia D (2003). Population genetic structure of mahogany (*Swietenia macrophylla* king, Meliaceae) across the Brazilian Amazon, based on variation at microsatellite loci: implications for conservation. Mol Ecol.

[51] Leo MD, Milella L, Braca A, Tommasi ND. Cedrela and Toona genera: a rich source of bioactive limonoids and triterpenoids. Phytochem Rev. 2018;17:751–783.

[52] Carpinella MC, Defago MT, Valladares G, Palacios SM (2003). Antifeedant and insecticide properties of a limonoid from *Melia azedarach* (Meliaceae) with potential use for pest management. J Agr Food Chem.

[53] Wandscheer CB, Duque JE, da Silva MA, Fukuyama Y, Wohlke JL, Adelmann J, et al. Larvicidal action of ethanolic extracts from fruit endocarps of *Melia azedarach* and *Azadirachta indica* against the dengue mosquito *Aedes aegypti*. Toxicon. 2004;44:829–35. 10.1016/j.toxicon.2004.07.009 15530964

[54] Krishnan NM, Pattnaik S, Jain P, Gaur P, Choudhary R, Vaidyanathan S (2012). A draft of the genome and four transcriptomes of a medicinal and pesticidal angiosperm *Azadirachta indica*. BMC Genomics.

[55] Brousseau L, Tinaut A, Duret C, Lang T, Garnier-Gere P, Scotti I (2014). High-throughput transcriptome sequencing and preliminary functional analysis in four Neotropical tree species. BMC Genomics.

[56] Zhang X, Song Z, Liu T, Guo L, Li X (2016). *De novo* assembly and comparative transcriptome analysis provide insight into lysine biosynthesis in *Toona sinensis* Roem. Int J Genomics.

[57] Tuskan GA, DiFazio S, Jansson S, Bohlmann J, Grigoriev I, Hellsten U (2006). The genome of black cottonwood, *Populus trichocarpa* (Torr. &amp; gray). Science.

[58] Sork VL, Fitz-Gibbon ST, Puiu D, Crepeau M, Gugger PF, Sherman R, et al. First draft assembly and annotation of the genome of a California endemic oak *Quercus lobata* Née (Fagaceae). G3: Genes Genom Genet. 2016;6:3485–95. 10.1534/g3.116.030411 PMC5100847 27621377

[59] Syring J, Cronn R, Tennessen JA, Jennings TN, Scelfo-Dalbey C, Wegrzyn J (2016). Targeted capture sequencing in whitebark pine reveals range-wide demographic and adaptive patterns despite challenges of a large, repetitive genome. Front Plant Sci.

[60] Neves LG, Davis JM, Barbazuk WB, Kirst M (2013). Whole-exome targeted sequencing of the uncharacterized pine genome. Plant J.

[61] Heyduk K, Trapnell DW, Barrett CF, Leebens-Mack J (2015). Phylogenomic analyses of species relationships in the genus *Sabal* (Arecaceae) using targeted sequence capture. Biol J of the Linn Soc.

[62] Weitemier K, Straub SC, Cronn RC, Fishbein M, Schmickl R, McDonnell A (2014). Hyb-Seq: combining target enrichment and genome skimming for plant phylogenomics. Appl Plant Sci.

[63] Ogden R, Dawnay N, McEwing R (2009). Wildlife DNA forensics—bridging the gap between conservation genetics and law enforcement. Endanger Species Res.

[64] Nicholls JA, Pennington RT, Koenen EJ, Hughes CE, Hearn J, Bunnefeld L (2015). Using targeted enrichment of nuclear genes to increase phylogenetic resolution in the neotropical rain forest genus *Inga* (Leguminosae: Mimosoideae). Front Plant Sci.

[65] Mandel JR, Dikow RB, Funk VA, Masalia RR, Staton SE, Kozik A (2014). A target enrichment method for gathering phylogenetic information from hundreds of loci: an example from the Compositae. Appli Plant Sci.

[66] Faircloth BC, McCormack JE, Crawford NG, Harvey MG, Brumfield RT, Glenn TC (2012). Ultraconserved elements anchor thousands of genetic markers spanning multiple evolutionary timescales. Syst Biol.

[67] Muellner AN, Pennington TD, Chase MW (2009). Molecular phylogenetics of Neotropical Cedreleae (mahogany family, Meliaceae) based on nuclear and plastid DNA sequences reveal multiple origins of “*Cedrela odorata*.”. Mol Phylogenet Evol.

[68] Zelener N, Tosto D, de Oliveira LO, Soldati MC, Inza MV, Fornes LF (2016). Molecular evidence of hybrid zones of *Cedrela* (Meliaceae) in the Yungas of northwestern Argentina. Mol Phylogenet Evol.

[69] Saeki I, Dick CW, Barnes BV, Murakami N (2011). Comparative phylogeography of red maple (*Acer rubrum* L.) and silver maple (*Acer saccharinum* L.): impacts of habitat specialization, hybridization and glacial history. J Biogeogr.

[70] Thomson AM, Dick CW, Dayanandan S (2015). A similar phylogeographical structure among sympatric north American birches (*Betula*) is better explained by introgression than by shared biogeographical history. J Biogeogr.

[71] Petit RJ, Kremer A, Wagner DB (1993). Geographic structure of chloroplast DNA polymorphisms in European oaks. Theor Appl Genet.

[72] Nevill PG, Després T, Bayly MJ, Bossinger G, Ades PK (2014). Shared phylogeographic patterns and widespread chloroplast haplotype sharing in *Eucalyptus* species with different ecological tolerances. Tree Genet Genomes.

[73] Premoli AC, Mathiasen P, Cristina Acosta M, Ramos VA (2012). Phylogeographically concordant chloroplast DNA divergence in sympatric *Nothofagus ss* how deep can it be?. New Phytol.

[74] rbpa: Transcriptome Assembly Pipeline BPA2.1.0. Shell. National Center for Genome Resources; 2017. https://github.com/ncgr/rbpa. Accessed 5 June 2018.

[75] Bolger AM, Lohse M, Usadel B (2014). Trimmomatic: a flexible trimmer for Illumina sequence data. Bioinformatics.

[76] Birol I, Jackman SD, Nielsen CB, Qian JQ, Varhol R, Stazyk G (2009). *De novo* transcriptome assembly with ABySS. Bioinformatics.

[77] Li W, Godzik A (2006). Cd-hit: a fast program for clustering and comparing large sets of protein or nucleotide sequences. Bioinformatics.

[78] Fu L, Niu B, Zhu Z, Wu S, Li W (2012). CD-HIT: accelerated for clustering the next-generation sequencing data. Bioinformatics.

[79] Huang X, Madan A (1999). CAP3: a DNA sequence assembly program. Genome Res.

[80] Luo R, Liu B, Xie Y, Li Z, Huang W, Yuan J (2012). SOAPdenovo2: an empirically improved memory-efficient short-read de novo assembler. Gigascience.

[81] Smith-Unna R, Boursnell C, Patro R, Hibberd J, Kelly S. TransRate: reference free quality assessment of de novo transcriptome assemblies. Genome Res. 2016;26:1–11. 10.1101/gr.196469.115 PMC4971766 27252236

[82] Smit AF (2004). Repeat-Masker Open-3.0.

[83] Tarailo-Graovac M, Chen N. Using RepeatMasker to identify repetitive elements in genomic sequences. Current Protoc Bioinformatics. 2004;5:4–10. 10.1002/0471250953.bi0410s25 19274634

[84] R. Core Team (2013). R: A language and environment for statistical computing.

[85] Li H, Durbin R (2010). Fast and accurate long-read alignment with burrows–wheeler transform. Bioinformatics.

[86] Li H. Aligning sequence reads, clone sequences and assembly contigs with BWA-MEM. arXiv preprint arXiv:13033997v2. 2013;00:1-3.

[87] Ovcharenko I, Loots GG, Giardine BM, Hou M, Ma J, Hardison RC, et al. Mulan: Multiple-sequence local alignment and visualization for studying function and evolution. Genome Res. 2005;15:184–94. 10.1101/gr.3007205 PMC540288 15590941

[88] Kearse M, Moir R, Wilson A, Stones-Havas S, Cheung M, Sturrock S (2012). Geneious basic: an integrated and extendable desktop software platform for the organization and analysis of sequence data. Bioinformatics.

[89] Maddison WP, Maddison DR (2017). Mesquite: a modular system for evolutionary analysis.

[90] Miller MA, Pfeiffer W, Schwartz T. The CIPRES science gateway: a community resource for phylogenetic analyses. In: Proceedings of the 2011 TeraGrid conference: extreme digital discovery, Salt Lake City, Utah, USA; 2011. p. 41.

[91] Rambaut A. FigTree, version 1.3. 1. Computer program distributed by the author, website: http://treebioedacuk/software/figtree/. Accessed 4 Jan 2011. 2009.

