# Supplementary material for: Genomic resources for the Neotropical tree genus Cedrela (Meliaceae) and its relatives
Source: BMC Genomics. 2019 Jan 18;20:58. doi: 10.1186/s12864-018-5382-6 (PMC6339301; doi:10.1186/s12864-018-5382-6)

**BMC Genomics Supporting Information**

**Abstract title: Genomic resources for the Neotropical tree genus *Cedrela* (Meliaceae) and its relatives**

**Authors: Kristen N. Finch, F. Andrew Jones, Richard C. Cronn**

**The following Supporting Information is available for this article:**

**Additional File 1 (this document): Table S1.** Specimen source and collection information. **Figure S1.** Distribution of Log_2_(Mapped Reads) for the gene models. **Figure S2.** Alternative view of main text Figure 3. **Figure S3.** Bootstrap consensus maximum likelihood species tree inferred from whole chloroplast genomes.

**Additional File 2: Table S2.** List of R packages used with version and citation.

**Finch, K. N. (2018). Dataset for genomic resources for the neotropical tree genus *Cedrela* (Meliaceae) and its relatives [Data set]. Oregon State University.** <https://doi.org/10.7267/NV935820Q>. Readers will find: the assembled transcriptome reference, hybridization capture probe sequences, the chloroplast genome reference for CEOD-NYBG, chloroplast genome sequences for each of the 43 specimens screened in our diversity panel (as separate files and as a combined file, aligned and unaligned), the VCF file containing SNPs for species and origin prediction for *Cedrela*, data sets to replicate our statistical analysis using R.

**Table S1.** Specimen source and collection information. Source abbreviations: Missouri Botanical Garden Herbarium (MO), New York Botanical Garden (NYBG), Fairchild Tropical Botanic Garden (FTBG), National Center for Biotechnology Information (NCBI), and fresh tissue collections from various locations across Panama (Panama).

| Specimen ID | Source | Country | Site/Plot | Latitude | Longitude | Year | Accession |
| --- | --- | --- | --- | --- | --- | --- | --- |
| *Azadirachta indica* | NCBI | India | NA | NA | NA | NA | NC_023792.1 |
| *Cedrela angustifolia* 88 | MO | Bolivia | NA | -21.41666 | -64.26666 | 1983 | 3446491 |
| *C. angustifolia* 143 | MO | Bolivia | NA | -21.0927777 | -64.2727778 | 2005 | 6268702 |
| *C. fissilis* 9 | MO | Bolivia | NA | -14.66861 | -62.60166 | 1992 | 4780122 |
| *C. fissilis* 19 | MO | Brazil | NA | -12.41666 | -64.23333 | 1987 | 5969966 |
| *C. fissilis* 112 | MO | Ecuador | NA | -0.03333 | -76.7 | 1986 | 4640039 |
| *C. fissilis* 130 | MO | Bolivia | NA | -15.05583 | -61.80333 | 1993 | 4910184 |
| *C. fissilis* 140 | MO | Bolivia | NA | -14.75583 | -61.04166 | 1995 | 5004112 |
| *C. fissilis* 211 | MO | Bolivia | NA | -15.24611 | -61.24277 | 1996 | 4826710 |
| *C. fissilis* 230 | MO | Bolivia | NA | -14.60361 | -61.49111 | 1997 | 5863117 |
| *C. fissilis* 254 | MO | Bolivia | NA | -19.8125 | -64.09972 | 2006 | 6050066 |
| *C. fissilis* 264 | MO | Bolivia | NA | -19.76666 | -63.975 | 1997 | 6032798 |
| *C. fissilis* 292 | MO | Bolivia | NA | -16.61666 | -67.5 | 1990 | 4989468 |
| *C. montana* 50 | MO | Colombia | NA | 4.01805 | -75.88027 | 1984 | 3252362 |
| *C. odorata*  CEOD-NYBG | NYBG | Mexico | NA | NA | NA | 1989 | 683/89 |
| *C. odorata* 10 | MO | Nicaragua | NA | 11.43333 | -85.55 | 1985 | 5807855 |
| *C. odorata* 52 | MO | Costa Rica | NA | 10.53333 | -85.3 | 1990 | 4311630 |
| *C. odorata* 162 | MO | Nicaragua | NA | 13 | -86.26666 | 1981 | 5879192 |
| *C. odorata* 185 | MO | Panama | NA | 8.5166667 | -82.7 | 1985 | 5807888 |
| *C. odorata* 202 | MO | Venezuela | NA | 9.6 | -72.9166667 | 1994 | 5000626 |
| *C. odorata* 222 | MO | Panama | NA | 9.15833 | -79.82222 | 1970 | 2029287 |
| *C. odorata* 277 | MO | Costa Rica | NA | 9.9 | -84.27 | 1997 | 4914056 |
| *C. odorata* 287 | MO | Colombia | NA | 6.53333 | -76.31666 | 1993 | 5027864 |
| *C. odorata* | NCBI | Cuba | NA | 20.16 | -76.68 | NA | NC_037251.1 |
| *C. saltensis* 75 | MO | Bolivia | NA | -21.1161111 | -64.2422222 | 2008 | 6403805 |
| *C. saltensis* 102 | MO | Bolivia | NA | -20.4002778 | -63.9297223 | 1999 | 5894370 |
| *C. saltensis* 186 | MO | Bolivia | NA | -22.2666667 | -64.5 | 1983 | 3446492 |
| *Guarea guidonia 2* | Panama | Panama | El Charco | NA | NA | 2013 | 823490 |
| *G. guidonia 4* | Panama | Panama | PLR | NA | NA | 2014 | PLR160 |
| *G. guidonia 7* | Panama | Panama | Gamboa | NA | NA | 2014 | GAM25 |
| *G. guidonia 9* | Panama | Panama | El Charco | NA | NA | 2013 | 623387 |
| *G. guidonia 10* | Panama | Panama | PLR | NA | NA | 2014 | PLR170 |
| *G. guidonia 11* | Panama | Panama | PLR | NA | NA | 2014 | 520820 |
| *G. guidonia 13* | Panama | Panama | Gamboa | NA | NA | 2014 | GAM18 |
| *G. guidonia 15* | Panama | Panama | PLR | NA | NA | 2014 | PLR169 |
| *G. guidonia 17* | Panama | Panama | Gamboa | NA | NA | 2014 | GAM23 |
| *G. guidonia 19* | Panama | Panama | El Charco | NA | NA | 2013 | 624393 |
| *Swietenia mahagoni 21* | FTBG | USA | Plot #197 | NA | NA | 2014 | 70262A |
| *S. mahagoni 22* | FTBG | USA | Plot #158 | NA | NA | 2014 | Removed |
| *Trichilia tuberculata 1* | Panama | Panama | PLR | NA | NA | NA | 34670 |
| *T. tuberculata 3* | Panama | Panama | Howard | NA | NA | 2013 | 103 |
| *T. tuberculata 6* | Panama | Panama | PLR | NA | NA | NA | 34686 |
| *T. tuberculata 8* | Panama | Panama | Howard | NA | NA | 2013 | 116 |
| *T. tuberculata 12* | Panama | Panama | PLR | NA | NA | 2013 | 44040 |
| *T. tuberculata 18* | Panama | Panama | PLR | NA | NA | NA | 515644 |
| *T. tuberculata 20* | Panama | Panama | Howard | NA | NA | 2013 | 113 |

**Figure S1.** Distribution of Log_2_(Mapped Reads) for the gene models of the transcriptome and the inverse depth index range of each gene model. The range of our 10,001 gene targets is outlined in red. The blue line is the distribution of RPK.


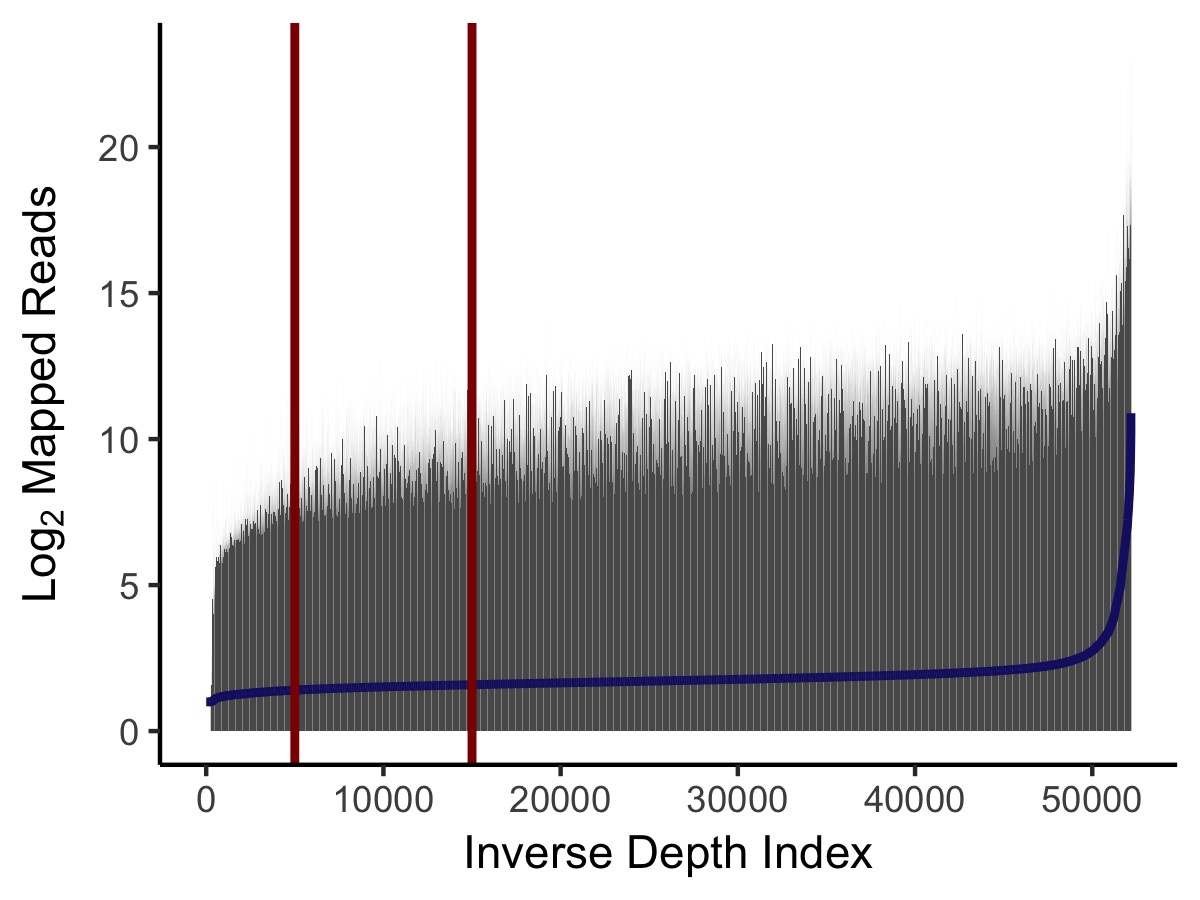


**Figure S2.** Violin plot showing depth of coverage (log_2_ scale) across enriched targets for each species. Mean depth of coverage for each species is indicated by a diamond. Distributions are color coded by species.


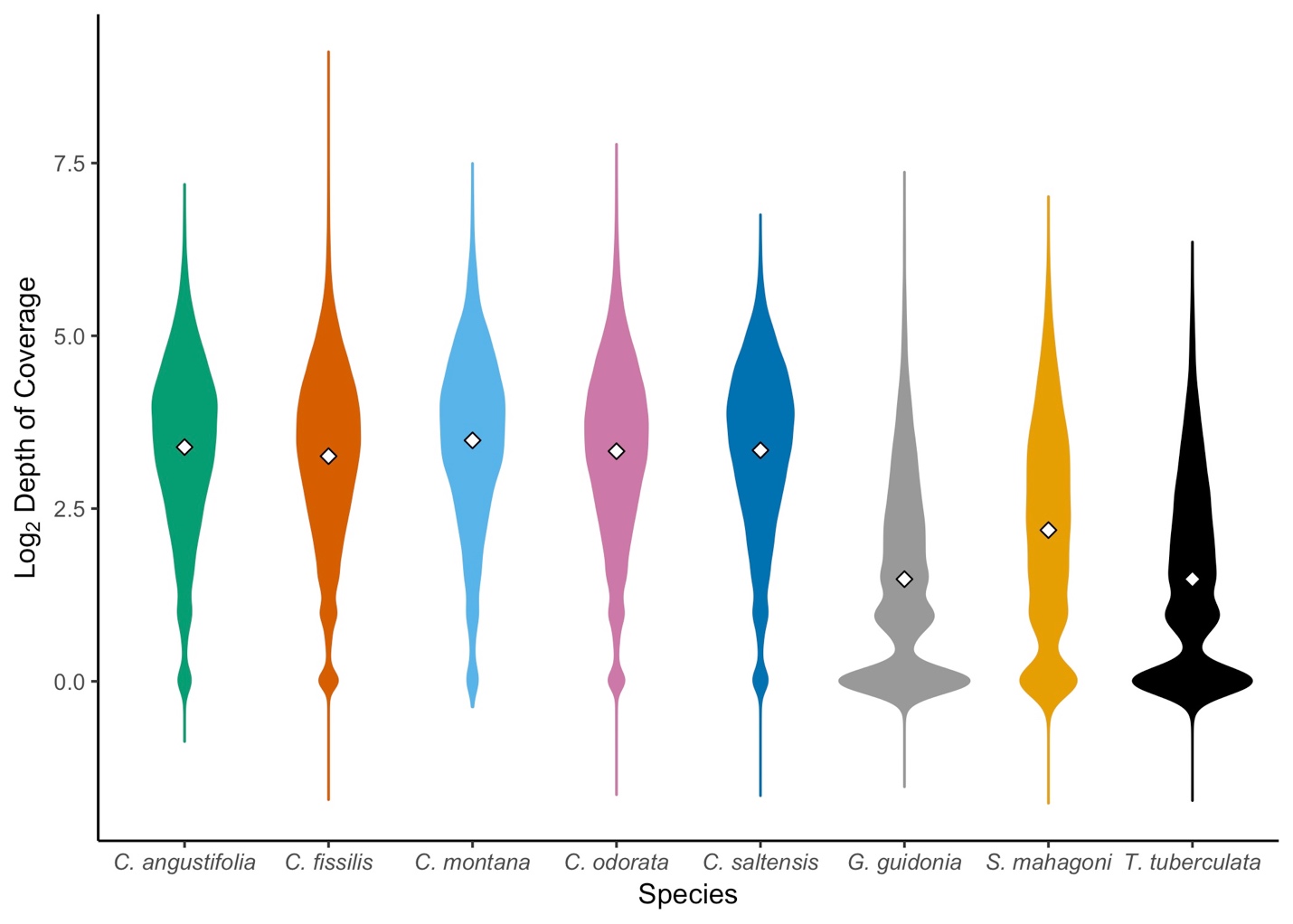


**Figure S3.** Bootstrap consensus maximum likelihood species tree inferred from whole chloroplast genomes. Taxa are color coded to match species in main text Fig. 3. Numbers near branches correspond to a subset of bootstrap support values for 1,000 bootstrap replicates.


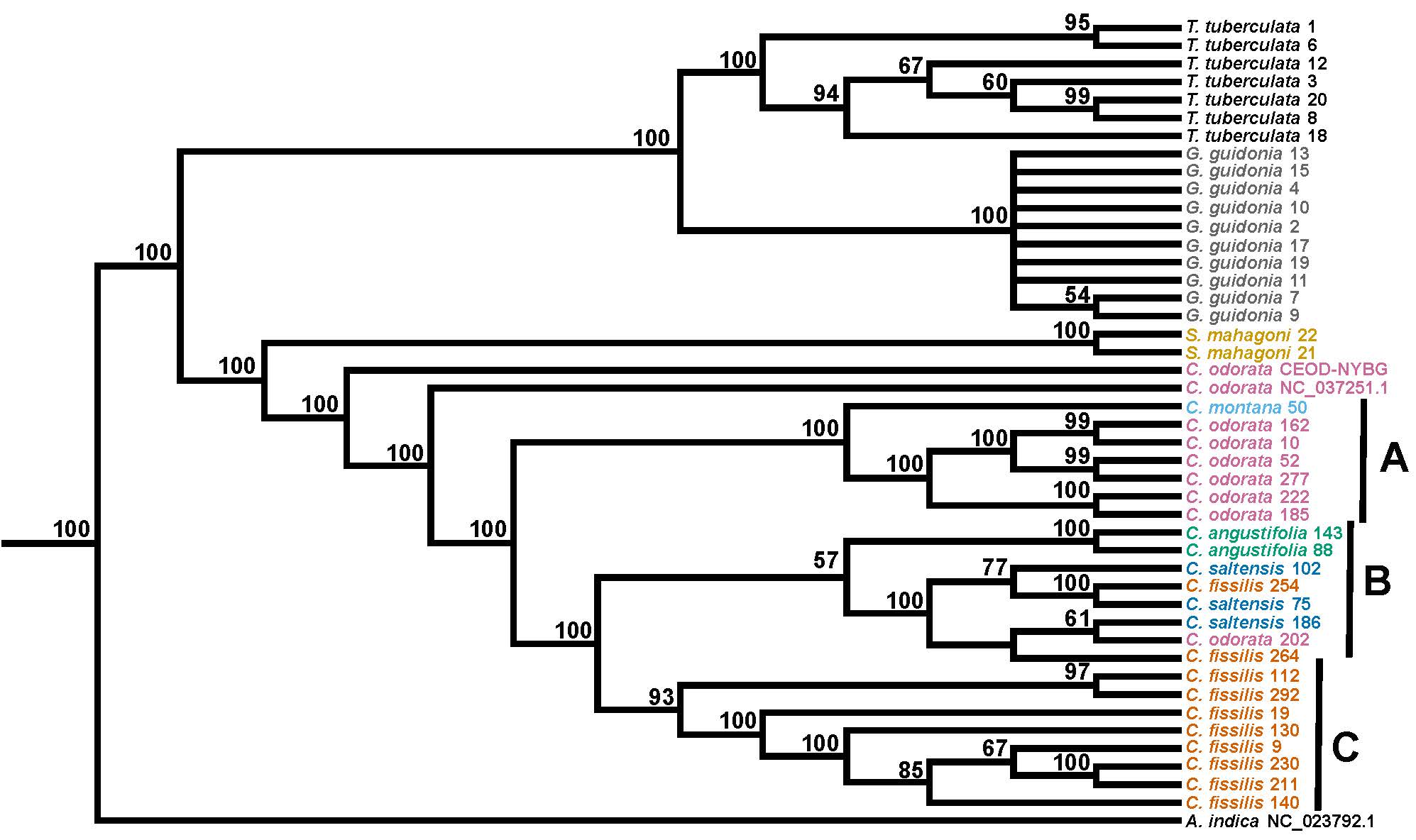

Supplement: Supplementary file 1 — Table S1. Specimen source and collection information. Figure S1. Distribution of Log2(Mapped Reads) for the gene models. Figure S2. Alternative view of main text Fig. 3. Figure S3. Bootstrap consensus maximum likelihood species tree inferred from whole chloroplast genomes. (DOCX 509 kb) [file 12864_2018_5382_MOESM1_ESM.docx]
