# Supplementary material for: Genomic resources for the Neotropical tree genus Cedrela (Meliaceae) and its relatives
Source: BMC Genomics. 2019 Jan 18;20:58. doi: 10.1186/s12864-018-5382-6 (PMC6339301; doi:10.1186/s12864-018-5382-6)
Supplement: Supplementary file 2 — Table S2. List of R packages used with version and citation. (DOCX 20 kb) Finch, K. N. (2018). Dataset for genomic resources for the neotropical tree genus Cedrela (Meliaceae) and its relatives [Data set]. Oregon State University. https://doi.org/10.7267/NV935820Q. Readers will find: the assembled transcriptome reference, hybridization capture probe sequences, the chloroplast genome reference for CEOD-NYBG, chloroplast genome sequences for each of the 43 specimens screened in our diversity panel (as separate files and as a combined file, aligned and unaligned), the VCF file containing SNPs for species and origin prediction for Cedrela, data sets to replicate our statistical analysis using R. (158 MB) [file 12864_2018_5382_MOESM2_ESM.docx]

**BMC Genomics Supporting Information**

**Abstract title: Genomic resources for the Neotropical tree genus *Cedrela* (Meliaceae) and its relatives**

**Authors: Kristen N. Finch, F. Andrew Jones, Richard C. Cronn**

**The following Supporting Information is available for this article:**

**Additional File 1: Table S1.** Specimen source and collection information. **Figure S1.** Distribution of Log_2_(Mapped Reads) for the gene models. **Figure S2.** Alternative view of main text Figure 3. **Figure S3.** Bootstrap consensus maximum likelihood species tree inferred from whole chloroplast genomes.

**Additional File 2 (this document): Table S2.** List of R packages used with version and citation.

**Finch, K. N. (2018). Dataset for genomic resources for the neotropical tree genus *Cedrela* (Meliaceae) and its relatives [Data set]. Oregon State University.** <https://doi.org/10.7267/NV935820Q>. Readers will find: the assembled transcriptome reference, hybridization capture probe sequences, the chloroplast genome reference for CEOD-NYBG, chloroplast genome sequences for each of the 43 specimens screened in our diversity panel (as separate files and as a combined file, aligned and unaligned), the VCF file containing SNPs for species and origin prediction for *Cedrela*, data sets to replicate our statistical analysis using R.

**Table S2.** R packages used for analysis.

| Package Citation | Package Name | Package Version |
| --- | --- | --- |
| [1] | dplyr | 0.7.6 |
| [2] | ggplot2 | 3.0.0 |
| [3] | ggrepel | 0.8.0 |
| [4] | gridBase | 0.4-7 |
| [5] | maps | 3.3.0 |
| [6] | maptools | 0.9-4 |
| [7] | reshape | 0.8-7 |
| [8] | rgdal | 1.3-4 |
| [9] | rgeos | 0.3-28 |
| [10] | scales | 1.0.0 |
| [11] | stringr | 1.3.1 |

**References obtained in R with citation() function.**

1. Hadley Wickham, Romain François, Lionel Henry and Kirill Müller (2018). dplyr: A Grammar of Data Manipulation. R package version 0.7.6. <https://CRAN.R-project.org/package=dplyr>

2. H. Wickham. ggplot2: Elegant Graphics for Data Analysis. Springer-Verlag New York, 2016.

3. Kamil Slowikowski (2018). ggrepel: Automatically Position Non-Overlapping Text Labels with 'ggplot2'. R package version 0.8.0. <https://CRAN.R-project.org/package=ggrepel>

4. Paul Murrell (2014). gridBase: Integration of base and grid graphics. R package version 0.4-7. <https://CRAN.R-project.org/package=gridBase>

5. Original S code by Richard A. Becker, Allan R. Wilks. R version by Ray Brownrigg. Enhancements by Thomas P Minka and Alex Deckmyn. (2018). maps: Draw Geographical Maps. R

package version 3.3.0. <https://CRAN.R-project.org/package=maps>

6. Roger Bivand and Nicholas Lewin-Koh (2018). maptools: Tools for Handling Spatial Objects. R package version 0.9-4. <https://CRAN.R-project.org/package=maptools>

7. H. Wickham. Reshaping data with the reshape package. Journal of Statistical Software, 21(12), 2007.

8. Roger Bivand, Tim Keitt and Barry Rowlingson (2018). rgdal: Bindings for the 'Geospatial' Data Abstraction Library. R package version 1.3-4. <https://CRAN.R-project.org/package=rgdal>

9. Roger Bivand and Colin Rundel (2018). rgeos: Interface to Geometry Engine - Open Source ('GEOS'). R package version 0.3-28. <https://CRAN.R-project.org/package=rgeos>

10. Hadley Wickham (2018). scales: Scale Functions for Visualization. R package version 1.0.0. <https://CRAN.R-project.org/package=scales>

11. Hadley Wickham (2018). stringr: Simple, Consistent Wrappers for Common String Operations. R package version 1.3.1. <https://CRAN.R-project.org/package=stringr>
